# LncRNA PVT1 promotes gemcitabine resistance of pancreatic cancer via activating Wnt/β-catenin and autophagy pathway through modulating the miR-619-5p/Pygo2 and miR-619-5p/ATG14 axes

**DOI:** 10.1186/s12943-020-01237-y

**Published:** 2020-07-29

**Authors:** Cefan Zhou, Changhua Yi, Yongxiang Yi, Wenying Qin, Yanan Yan, Xueying Dong, Xuewen Zhang, Yuan Huang, Rui Zhang, Jie Wei, Declan William Ali, Marek Michalak, Xing-Zhen Chen, Jingfeng Tang

**Affiliations:** 1 National “111” Center for Cellular Regulation and Molecular Pharmaceutics, Key Laboratory of Fermentation Engineering (Ministry of Education), Hubei University of Technology, 28 NanLi Road, Wuhan, 430068 Hubei China; 2 Nanjing Clinical Medical Center for Infectious Diseases, the Second Affiliated Hospital of Southeast University (the Second Hospital of Nanjing), Nanjing, China; 3 Department of Biological Sciences, University of Alberta, Edmonton, Alberta Canada; 4 Department of Biochemistry, University of Alberta, Edmonton, Alberta Canada; 5 Membrane Protein Disease Research Group, Department of Physiology, Faculty of Medicine and Dentistry, University of Alberta, Edmonton, AB Canada

**Keywords:** PVT1, Gemcitabine resistance, Autophagy, miR-619-5p, Wnt/β-catenin

## Abstract

**Background:**

Pancreatic cancer is one of the most lethal malignancies and has an extremely poor diagnosis and prognosis. The development of resistance to gemcitabine is still a major challenge. The long noncoding RNA PVT1 was reported to be involved in carcinogenesis and chemoresistance; however, the mechanism by which PVT1 regulates the sensitivity of pancreatic cancer to gemcitabine remains poorly understood.

**Methods:**

The viability of pancreatic cancer cells was assessed by MTT assay in vitro and xenograft tumor formation assay in vivo. The expression levels of PVT1 and miR-619-5p were detected by quantitative real-time polymerase chain reaction (qRT-PCR). Western blotting analysis and qRT-PCR were performed to assess the protein and mRNA levels of Pygo2 and ATG14, respectively. Autophagy was explored via autophagic flux detection under confocal microscopy and autophagic vacuole investigation under transmission electron microscopy (TEM). The functional role and mechanism of PVT1 were further investigated by gain- and loss-of-function assays in vitro.

**Results:**

In the present study, we demonstrated that PVT1 was up-regulated in gemcitabine-resistant pancreatic cancer cell lines. Gain- and loss-of-function assays revealed that PVT1 impaired sensitivity to gemcitabine in vitro and in vivo. We further found that PVT1 up-regulated the expression of both Pygo2 and ATG14 and thus regulated Wnt/β-catenin signaling and autophagic activity to overcome gemcitabine resistance through sponging miR-619-5p. Moreover, we discovered three TCF/LEF binding elements (TBEs) in the promoter region of PVT1, and activation of Wnt/β-catenin signaling mediated by the up-regulation of Pygo2 increased PVT1 expression by direct binding to the TBE region. Furthermore, PVT1 was discovered to interact with ATG14, thus promoting assembly of the autophagy specific complex I (PtdIns3K-C1) and ATG14-dependent class III PtdIns3K activity.

**Conclusions:**

These data indicate that PVT1 plays a critical role in the sensitivity of pancreatic cancer to gemcitabine and highlight its potential as a valuable target for pancreatic cancer therapy.

## Background

Pancreatic cancer is one of the most lethal malignancies due to its frequently late diagnosis, aggressive tumor growth and high level of metastasis. The overall 5-year survival rate of patients with pancreatic cancer is less than 5% [1]. Due to the low resection rate and high recurrence rate of pancreatic cancer, chemotherapy plays an important role in the comprehensive treatment of pancreatic cancer. Gemcitabine (2′,2 ‘-difluoro deoxycytidine (dFdC)) is a first-line chemotherapy drug approved for the treatment of advanced pancreatic cancer, either alone or in combination with other chemotherapeutic agents [2]. Although in recent clinical trials combinations of gemcitabine with other drugs resulted in relative improvements in median survival time, unfortunately, gemcitabine failed to significantly improve the overall prognosis and survival rates of pancreatic cancer patients, which was mainly caused by natural and/or acquired resistance to gemcitabine [3]. Thus, the inherent resistance of pancreatic cancer to currently available chemotherapeutic agents presents a major challenge. Therefore, the identification of genetic determinants of sensitivity to chemotherapeutic agents is imperative for the development of effective treatments and biomarkers for pancreatic cancer.

Long noncoding RNAs (lncRNAs) comprise a heterogeneous family of RNA molecules longer than 200 nucleotides with no or limited protein-coding potential. LncRNAs mainly play their roles through the following mechanisms: 1) LncRNAs competitively adsorb miRNA as competing endogenous RNAs (ceRNAs) through base complementarity, resulting in the loss or reduction of miRNA function and controlling the regulation of miRNAs on target genes. 2) LncRNAs activate or inhibit certain cell activities by binding some epigenetic-related proteins to modify specific protein translation after transcription. 3) LncRNAs inhibit gene transcription by directly binding chromosomal DNA or promote gene transcription by recruiting transcription factors. 4) Finally, lncRNAs function as precursors of miRNAs [4]. Recent findings have highlighted the interaction of lncRNAs with cancer and chemotherapeutic resistance. For example, the lncRNAs HOTAIR and LINC00346 were reported to drive castration resistance in prostate cancer and promote pancreatic cancer growth and gemcitabine resistance, respectively [5]. The lncRNA human plasmacytoma variant translocation1 (PVT1), which is located downstream of the human chromosome 8q24 and proto-oncogene C-Myc, is highly expressed in a variety of tumor tissues or cell lines [6]. High PVT1 expression in these tumors is indicative of poor prognosis. Various studies have revealed that PVT1 plays important roles in carcinogenesis and chemoresistance [7]. Additionally, functional inactivation of the PVT1 gene could enhance gemcitabine sensitivity, whereas ectopic expression of PVT1 reversed this effect in ASPC-1 human pancreatic cancer cells [8]. However, the functional roles and mechanisms of PVT1 in the sensitivity of pancreatic cancer to gemcitabine treatment remain poorly understood and thus need to be further clarified.

Macroautophagy (hereafter referred to as autophagy) is an evolutionarily ancient and highly conserved catabolic process. It begins with the formation of double-membrane vesicles known as autophagosomes that engulf cellular proteins and organelles for delivery to the lysosome [9]. Autophagy requires the involvement of several autophagy-related (ATG) proteins in response to nutrient depletion and subsequent adenosine 5′-monophosphate (AMP)-activated protein kinase (AMPK) activation or mechanistic target of rapamycin kinase (mTOR) inhibition [10, 11]. Autophagy is one of the mechanisms by which solid tumors resist hypoxia, ischemia and other harsh environments [12]. Autophagy has been shown to promote the tolerance of tumor cells to radiotherapy and chemotherapy [13, 14]. Blockade of autophagy with chloroquine also significantly increased the sensitivity of pancreatic cancer cells to gemcitabine [15].

The Wnt/β-catenin signaling pathway plays an important role in regulating various cellular activities, such as self-renewal, cell proliferation, embryonic development, tissue homeostasis and cell death [16]. Intracellular signal transduction by the Wnt/β-catenin signaling pathway is triggered when Wnt ligand proteins bind receptors of the Frizzled and LRP families on the cell surface. Through several cytoplasmic relay components, the signal is transduced to β-catenin, which enters the nucleus and forms a complex with TCF to activate the transcription of Wnt target genes [16]. Several studies have revealed that the activity of the Wnt/β-catenin signaling pathway could be induced and elevated under the conditions of chemotherapy [17]. Furthermore, pharmacological or genetic inhibition of Wnt activity restored the chemosensitivity of cancer cells, even in mouse models [18].

In this study, we attempted to investigate the contributions and mechanisms of the lncRNA PVT1 to the sensitivity of pancreatic cancer to gemcitabine. We demonstrated that PVT1 specifically promotes pancreatic cancer cell resistance to gemcitabine via increased Wnt/β-catenin pathway signaling and autophagic activity. We also indicated that PVT1 modulates gemcitabine resistance by the miR-619-5p/Pygo2 and miR-619-5p/ATG14 axes in pancreatic cancer cells. Moreover, we found that PVT1 is up-regulated by Pygo2 through increased binding of the β-catenin/TCF4 complex to its promoter region. Furthermore, we also showed that PVT1 interacts with ATG14 in the cytoplasm and promotes assembly of the PtdIns3K-C1 complex and the activation of ATG14-dependent class III PtdIns3K activity. Collectively, our findings highlight the role of PVT1 in the regulation of chemoresistance in pancreatic cancer.

## Methods

### Cell lines, reagents, and antibodies

Human pancreatic cancer cell lines PANC-1 and ASPC-1 were purchased from the cell center of Institute of Biochemistry and Cell Biology, Chinese Academy of Sciences (TCHu 98 and TCHu 8). The human pancreatic cancer cell line SW1990 and gemcitabine resistant cell line SW1990/GEM was purchased from Biobw (Beijing, China, bio-106,239). HEK293T cell line was maintained in our lab. Gemcitabine-resistant PANC-1 (PANC-1/Gem) cells were established by our laboratory. Briefly, PANC-1 cells were initially exposed to 0.2 μM of gemcitabine for 1 week. When cells returned to normal growth rate after the period of recovery, the concentrations of gemcitabine were gradually increased (5, 10, 20 and 40 μM) until cells became resistant to 40 μM of gemcitabine, approximately for 10 months. Gemcitabine HCl was purchased from APEXBIO (A1402). Nocodazole and autophagy inhibitor chloroquine diphosphate salt (CQ, 10 μM) was purchased from Sangon Biotech (A606391 and A506569). Wnt/β-catenin inhibitor XAV-939 was purchased from Selleck (S1180). Wnt/β-catenin activator lithium chloride (LiCl) and recombinant mouse Wnt3a protein were purchased from Sangon Biotech (A100416) and Abcam (ab81484) respectively. Commercially available antibodies and dilutions used are as follows: anti-β-catenin (Proteintech, 51,067–2-AP; 1:1000 dilution), anti-CyclinD1 (Proteintech, 60,186–1-Ig; 1:1000 dilution), anti-C-myc (Proteintech, 60,178–1-Ig; 1:1000 dilution), anti-Axin2 (Proteintech, 20,540–1-AP; 1:1000 dilution), anti-Ki67 (BOSTER, BM4381; 1:1000 dilution), anti-GAPDH (Proteintech, 60,004–1-Ig; 1:1000 dilution), anti-LC3 (Cell Signaling Technology, 4108; 1:1000 dilution), anti-p62/SQSTM1 (Proteintech, 18,420–1-AP; 1:1000 dilution), anti-Mouse HA (EMD Millipore, M180–3; 1:1000 dilution), anti-Rabbit HA (Proteintech, 51,064–2-AP; 1:1000 dilution), anti-Pygo2 (Santa Cruz, sc-390,506, 1:1000 dilution), anti-P-Glycoprotein/P-gp (Proteintech, 22,336–1-AP; 1:1000 dilution), anti-Ago2 (Proteintech, 10,686–1-AP; 1:1000 dilution), anti-ATG14/Barkor (N-Terminal) (Proteintech, 19,491–1-AP; 1:1000 dilution), anti-Beclin1/BECN1 (Proteintech, 11,306–1-AP; 1:1000 dilution), anti-PIK3C3/VPS34 (Proteintech, 12,452–1-AP; 1:1000 dilution). anti-ATG7 (Proteintech, 67,341–1-Ig; 1:1000 dilution). anti-ATG9 (Proteintech, 26,276–1-AP; 1:1000 dilution). anti-PIK3C3/VPS34 (Proteintech, 12,452–1-AP; 1:1000 dilution). anti-ULK1 (Boster, BM5170; 1:500 dilution).

### Plasmids construction, cell culture, siRNAs and transfection

DNA fragments encoding LncRNA PVT1 and Pygo2 were amplified by PCR from the genomic DNA of PANC-1 cells, and sub-cloned into the pCDNA3.0 and pCMV-HA plasmid respectively. DNA fragments encoding ATG14 and ZFYVE1 amplified by PCR were gifts from Prof. Jiahuai Han (Xiamen University, China). The PCR product of ATG14 was cloned into pCMV-HA. The PCR products of ZFYVE1 was cloned into pCMV-N1-EGFP (Clontech, 6085–1). GFP-LC3 was a gift from Prof. QingKenneth Wang (Huazhong University of Science and Technology, China). The nucleotide sequences of all constructs were confirmed by DNA sequencing. The sequence of miR-619-5p was 5′ - GCUGGGAUUACAGGCAUGAGCC-3′. Commercially synthesized 2′ -O-methyl-modified antisense oligonucleotide of miR-619-5p was used as a miR-619-5p inhibitor. HEK293T cell line and human pancreatic cancer cells PANC-1, ASPC-1 and SW1990 were cultured in Dulbecco’s modified Eagle’s medium (DMEM; Hyclone, SH30022.01). All culture media were supplemented with 10% fetal bovine serum (Biological Industries, 04–001-1ACS), 100 U/ml penicillin G and 100 μg/ml (Biosharp, BL505A) streptomycin at 37 °C in a humidified incubator containing 5% CO2. siRNA for PVT1#1 was 5′- GAGCUGCGAGCAAAGAUGU-3′, #2 was 5′- ACUUUAAGUGGAGGCUGA AUCAUCU − 3′. siRNA for Pygo2 was 5′- CCAUGAUCUCACCCACCAU − 3′. siRNA for ATG14 was 5′- AUCUUCGACGAUCCCAUAUAUUA − 3′. siRNA for ATG7 was 5′- CAGCCUGGCAUUUGAUAAATT − 3′. Lipofectamine 2000 Transfection Reagent (Invitrogen, 11,668,019) was used to transfect the PANC-1, ASPC-1 and SW1990 cell lines with the miR-619-5p mimics, inhibitors and siRNAs, and the expression vector of PVT1, Pygo2 or ATG14 was transfected using Lipofectamine 3000 (Invitrogen, L3000015) according to the manufacturer’s protocols.

### RNA extraction and qRT-PCR

RNA extraction and qRT-PCR were previously described [19]. Total RNA was extracted from cultured pancreatic cancer cells using Trizol reagent (Invitrogen, 15596026) according to the manufacturer’s protocol. RNAs were quantified using a NanoDrop 2000c instrument (ThermoFisher). cDNA was reverse transcribed using a HiScript cDNA synthesis kit (Vazyme, R323–01). Quantitative PCR was performed using the Universal SYBR qPCR Master Mix (Vazyme, Q511–02). The mRNA expression level for each sample was normalized to the expression of GAPDH and miRNA expression was normalized to RNU6B using the 2 ^-ΔΔct^ method [20] with three biological replicates of comparative qRT-PCR. The following primer sequences were used for qRT-PCR: PVT1, (forward) 5′-CCTGGTGAAGCATCTGATGCACG-3′ and (reverse) 5′-GCCAGGCTTTGTGGCACACGC-3′; Pygo2, (forward) 5′-CCTGCATACTCACATCTGACGGAGT-3′ and (reverse) 5′-CTGCCCACTGGGAGGACTAAAG-3′; ATG14, (forward) 5′-CGTCTACTTCGACGGCCGCGA-3′ and (reverse) 5′-CTCTTGGTGCCGTTGTGCTCG-3′; GAPDH, (forward) 5′-AGCCACATCGCTCAGACAC-3′ and (reverse) 5′-GCCCAATACGACCAAATCC-3′; CyclinD1, (forward) 5′-GCGTGTAGCTATGGAAGTTGCA-3′ and (reverse) 5′-CATCCCGAATGAGAGTCCTACAG-3′; C-myc, (forward) 5′-ATCTCACAGTGACCAACCCAAA-3′ and (reverse) 5′-TCGGTCACGGAGCCAATC-3′; Axin2, (forward) 5′-CAAGGGCCAGGTCACCAA-3′ and (reverse) 5′-CCCCAACCCATCTTCGT-3′; miR-619-5p-RT, 5′-GTCGTATCCAGTGCAGGGTCCGAGGTATTCGCACTGGATACGACGGCTCA-3′, (forward) 5′-CGGCTGGGATTACAGGCA-3′ and (reverse) 5′-AGTGCAGGGTCCGAGGTATT-3′; RNU6B, (forward) 5′-CTCGCTTCGGCAGCACA-3′ and (reverse) 5′-AACGCTTCACGAATTTGCGT-3′. U1, (forward) 5′-GGCGAGGCTTATCCATTG-3′ and (reverse) 5′-CCCACTACCACAAATTATGC-3′. Mouse Pvt1: (forward) 5′-TGTGAAGCGTTGACTTAAGAG-3′ and (reverse) 5′-GGCTGGATCTATCACCTG-3′; mouse Gapdh: (forward) 5′-CGAGAATGGGAAGCTTGTC-3′ and (reverse) 5′-CAAAGTTGTCATGGATGACC-3′.

### Lentiviral production and creation of stable cell lines

PVT1 shRNA or scramble RNA was subcloned into the lentiviral vector pLKO.1-puro (Sigma, 8453). The DNA fragment of PVT1 was subcloned into the lentiviral vector pCDH-CMV-MCS-EF1-turboRFP-T2A-Neo. The lentiviral constructs (5 μg) were co-transfected with the viral packaging plasmids psPAX2 (3 μg) and pMD2.G (3 μg) (both were gifts from Dr. Xiaorong Zhang [Institute of Biophysics, Chinese Academy of Sciences, China]) into HEK293T cells in 10 cm dishes. The viral supernatant was harvested at 48 h post-transfection, and passed through a 0.22 μm filter. After applying the viral supernatant to PANC-1, PNAC-1/Gem, SW1990 and SW1990/Gem cells with 10 μg/μl polybrene (Solarbio, H8761), the cells were selected with puromycin (Solarbio, IP1280) and/or G418 (Yeasen, 60220ES03) at 48 h after transfection. The selection medium was changed every 3–4 days for several weeks, and clones of puromycin- and/or G418- resistant cells were isolated and expanded for further characterization. Cells stably expressing PVT1 shRNA and PVT1 were maintained in complete culture medium with 2 μg/ml puromycin and/or 100 μg/ml G418.

### Dual-luciferase reporter assay

To assess the regulatory effects of PVT1 on miR-619-5p and miR-619-5p on Pygo2 and ATG14 mRNA, dual-luciferase reporter assay was performed as described previously [21]. Briefly, the PVT1 sequence containing 130–138 bp and the 3′UTR of Pygo2 containing 1748–1755 bp and ATG14 mRNA containing 1127–1133 bp and 1262–1268 bp were amplified from pancreatic cancer PANC-1 cells by PCR and cloned between the *XhoI* and *SalI* sites of the pMIRGLO dual-luciferase miRNA target expression vector (Promega, E1330). The primer sequences specific to PVT1 used for the dual-luciferase reporter assay were (forward) 5′-CCCTTTGAGCTGCTTGGCAC-3′ and (reverse) 5′-CTTGAGGTCAGGAGTTCGAGACC-3′. The Pygo2 3′UTR qRT-PCR primer sequences were (forward) 5′-CCCTCTCGCTCTCTCACTCCAC-3′ and (reverse) 5′-CCCTAAGCACCCTACCCAGC-3′. The ATG14 3′UTR qRT-PCR primer sequences were (forward) 5′-CTGATGCTACTCTGCTCTGTTCTGGG-3′ and (reverse) 5′-GAGACGGAGTTTCGCTCTTGTTGC-3′. The miR-619-5p target site-mutation of PVT1, Pygo2 and ATG14 3’UTR luciferase reporter construct was generated using the QuikChange II XL Site-Directed Mutagenesis Kit (Stratagene, 200,521). The nucleotide sequences of all constructs were confirmed by DNA sequencing. The ratio of experimental reporter (Firefly luciferase) luminescence to control reporter (Renilla luciferase) luminescence was calculated. All experiments were performed in triplicate.

To study the regulatory effect of β-catenin on the PVT1 promoter, the PVT1 promoter region from 2000 bp upstream to 1 bp downstream of the transcription start site (TSS) was cloned into plasmid pGL3-Basic firefly luciferase reporter plasmid (Promega, E1751). A Renilla luciferase vector was used as an internal control and purchased from Promega (E6971). The primer sequences specific to the PVT1 promoter used for qRT-PCR were (forward) 5′-CAGCATCAAGGTCAAAGTTGAGTGAGTCC-3′ and (reverse) 5′-CTCGGCCGCCACACGC-3′. The nucleotide sequences of all constructs were confirmed by DNA sequencing. The site-mutation or truncation of the PVT1 promoter region luciferase reporter construct was generated using overlap extension PCR assay or with a QuikChange II XL Site-Directed Mutagenesis Kit (Stratagene, 200,521). The ratio of experimental reporter (Firefly luciferase) luminescence to control reporter (Renilla luciferase) luminescence was calculated. All experiments were performed in triplicate.

### Cytosolic and nuclear fractionation

PANC-1 and ASPC-1 cells were washed with phosphate-buffered saline (PBS; Servicebio, WGSH30256–01) twice and incubated with hypotonic buffer (25 mM Tris-HCl, pH 7.4, 1 mM MgCl2, 5 mM KCl and 1% NP-40) on ice for 10 min. The supernatant of the cell lysates were collected as the cytoplasmic fraction at 5000×g for 5 min. Then the pellets were resuspended in nucleus resuspension buffer (20 mM HEPES, pH 7.9, 400 mM NaCl, 1 mM EDTA, 1 mM EGTA, 1 mM DTT, 1 mM PMSF) for 30 min. After centrifugation at 12,000×g for 10 min, the supernatant was collected as the nuclear fraction. Cytoplasmic and nuclear fractions were divided for RNA extraction. GAPDH and U1 were used as qRT-PCR markers of cytoplasmic and nuclear RNAs, respectively.

### TOP/FOP flash assay

The TOP/FOP Flash luciferase assay was performed as previously described [22]. M50 Super 8x TOPFlash and M51 Super 8x FOPFlash (TOPFlash mutant) were gifts from Randall Moon (Addgene, 12,456 and 12,457). PANC-1 human pancreatic cancer cells were transfected with the M50 and M51 plasmids along with an internal Renilla control plasmid. The luminescence ratio of the experimental reporter (Firefly) to control reporter (Renilla) was calculated. All experiments were performed in triplicate.

### RNA pull-down assays

Full-length sense and antisense PVT1 sequences were obtained using an in vitro transcription kit (ThermoFisher Scientific, K0441). RNA pull-down assays were performed using a Magnetic RNA-Protein Pull-down Kit (ThermoFisher Scientific, 20,164) according to the manufacturer’s instructions. Briefly, a single desthiobiotinylated cytidine bisphosphate was first attached to the 3′ ends of the full-length sense and antisense RNA PVT1 sequences. Then, 50 pmol of labeled RNA was added to 50 μl of streptavidin magnetic beads and incubated in RNA capture buffer (20 mM Tris [pH 7.5], 1 M NaCl, 1 mM EDTA) for 20 min at room temperature. After equilibrating the RNA-bound beads with protein-RNA binding buffer (0.2 M Tris [pH 7.5], 0.5 M NaCl, 20 mM MgCl_2_, 1% Tween-20), PANC-1 and ASPC-1 cell lysates were added to the beads and incubated for 2 h at 4 °C on a rotator. The beads were washed twice and incubated with elution buffer, and the supernatants were subjected to Western blotting assays.

### RNA immunoprecipitation (RIP) assay

PANC-1 and ASPC-1 cells were washed twice with PBS and lysed with RIPA lysis buffer (50 mM Tris-HCl [pH 7.4], 150 mM NaCl, 1% Triton X-100, 10 mM NaF, 1 mM EDTA, 100 U/ml RNaseOUT [ThermoFisher Scientific]) containing a proteinase inhibitor cocktail. The lysates were then incubated with magnetic beads conjugated with human anti-Ago2 antibody, anti-ATG14 antibody and normal rabbit IgG control. The beads were washed twice with a high salinity washing buffer (700 mM NaCl). RNA in the immunoprecipitate was isolated with TRIzol reagent (ThermoFisher Scientific, 15,596,018) and analyzed by qRT-PCR.

### Immunoprecipitation

PANC-1 and ASPC-1 cells were washed twice with PBS and lysed with RIPA lysis buffer (50 mM Tris-HCl [pH 7.4], 150 mM NaCl, 1% Triton X-100 [Sangon Biotech, 9002-93-1], 10 mM NaF, 1 mM EDTA) containing a proteinase inhibitor cocktail (Bimake, B14001) and Halt phosphatase inhibitor cocktail (Thermofisher Scientific, 78,420). The protein concentration was measured using the BioRad protein assay kit (BioRad, 5,000,006). Cell lysates were incubated overnight with primary antibodies depending on the experiment after pretreatment with IgG and protein A/G magnetic beads (Bimake, B23202) and then incubated with protein A/G magnetic beads for 2 h at 4 °C. The immunoprecipitates were then subjected to western blotting.

### Western blotting

Cell lysates were prepared as described previously [23]. Briefly, cell lysates or immunoprecipitates were heated in 2 × SDS loading buffer (100 mM Tris-HCl [pH 6.8], 4% [wt:vol] SDS, 200 mM dithiothreitol, 0.2% [wt:vol] bromophenol blue, and 20% [vol:vol] glycerol) for 5–10 min at 98 °C. Proteins were separated by SDS-PAGE gels and transferred to 0.45 μm polyvinylidene fluoride (PVDF) membranes (Millipore, IPFL85R). After the PVDF membranes had been blocked with TBS-T containing 5% skimmed milk at room temperature for 1 h and incubated with primary antibodies and secondary antibodies, the protein signals in the PVDF membranes were detected using SuperSignal West Pico PLUS (Invitrogen, 34,580) according to the manufacturer’s instructions.

### Immunohistochemistry

Immunohistochemistry was performed as described previously [21]. Briefly, antibodies against Ki67, CyclinD1, C-myc and p62 were tested on sections from excised xenograft tumor tissues.

### Cell viability assay

Cell viability was determined by MTT assay performed as described previously [24]. Briefly, PANC-1, PANC-1/Gem, SW1990 and SW1990/Gem cells (1 × 10^3^ cells/well) were seeded into 96- well plates. After 12 h of culture, the cells were treated with gemcitabine at the indicated concentration for another 24 h. The cells were then stained with 100 μl of sterile MTT dye (0.5 mg/ml; Sigma, M2128) for 4 h at 37 °C, followed by removal of the culture medium and the addition of 150 μl of DMSO (Sigma, W387520). The number of viable cells was assessed by measurement of the absorbance at 450 nm with a microplate reader. All experiments were performed in triplicate. To calculate the half inhibitory concentration (IC50), data were fitted in GraphPad Prism 6.0, and the dose-response curve was plotted using the equation log (inhibitor) vs. response- variable slope. The IC50 was obtained using the following formula: Y=Bottom + (TopBottom)/(1 + 10^((Log IC50-X) *HillSlope)).

### Colony formation assay

PANC-1 and ASPC-1 human pancreatic cancer cells transfected with PVT1 or miR-619-5p were seeded into a 12-well plate and incubated with complete medium at 37 °C for 2–3 weeks. Then, the cells were fixed with 4% paraformaldehyde and stained with 2% crystal violet. Images were obtained and the number of colonies was counted.

### Immunofluorescence and confocal microscopy

PANC-1 and ASPC-1 cells transfected with GFP-LC3, GFP-ZFYVE1 and another co-transfected overexpression or knockdown plasmids were grown on 12-well plates, and cells cultured for 48 h at 60% density were used for confocal microscopy on glass chambers. After the cells were fixed and stained with DAPI (Solarbio, C0065), images were obtained with a confocal laser-scanning microscope (Leica SP8, Wetzlar, Germany) using a 63× oil immersion objective. Data analysis was performed using Leica LAS AF Lite software. The numbers of GFP-LC3 and GFP-ZFYVE1 puncta per cell were assessed in 10 non-overlapping fields.

### TUNEL assay

Apoptotic cells among PANC-1 and ASPC-1 cells after gemcitabine treatment with or without XAV-939 (a Wnt inhibitor) or CQ (an autophagy inhibitor) treatment were examined with the One Step TUNEL (TdT-mediated dUTP nick-end labeling) Apoptosis Assay Kit (Beyotime, C1090) following the manufacturer’s protocols. Cells were photographed under an Olympus FSX100 microscope (Tokyo, Japan).

### Determination of caspase activity

The activity of caspase-3/7 was measured as described previously [19]. Briefly, cells were collected after treatment with gemcitabine for 48 h, after adding 100 μl of reaction mixture (100 mM HEPES, 10% sucrose, 0.1% CHAPS, 10 mM DTT, 1 mM EDTA [pH 7.4]) containing appropriate dilutions of the enzyme, pro-luminescent caspase-3 DEVD-amino luciferin substrate (Ac-DEVD-AFC). The related caspase activity was quantified by detection of the fluorescence of free AFC after cleavage from the peptide substrate using a fluorometer with ex. = 400 nm and em. = 505 nm.

### 5-Ethynyl-20-deoxyuridine (EdU) incorporation assay

The EdU assay was performed as described previously [24]. EdU labeled PANC-1 human pancreatic cancer cells transfected with PVT1 after gemcitabine treatment with or without the combination of Wnt inhibitor XAV-939 or autophagy inhibitor CQ treatment were examined with the BeyoClick™ EdU Cell Proliferation Kit with Alexa Fluor 555 (Beyotime, C0075S). Cells were photographed under an Olympus FSX100 microscope.

### Chromatin immunoprecipitation assay (ChIP)

ChIP assay was performed as described previously [22]. Briefly, cross-linked chromatin and protein complexes perpared using 1% formaldehyde were sonicated into ~ 500 bp fragments. The soluble material was then purified by centrifugation and mixed with polyclonal antibodies against TCF4 or β-catenin or control rabbit IgG overnight at 4 °C, followed by incubation with magnetic protein A/G beads under reverse rotation at 4 °C for 2 h. qPCR was used to analyze binding of the β-catenin/TCF-4 complex to the PVT1 promoter. The primers used were as follows: #1 (− 1800 ~ − 2000) (forward), 5′-GCCAGGGGAGATTTTCCATG-3′ and (reverse) 5′-CGATAGGAGCTGTGAGACCT-3′; #2 (− 1600 ~ − 1800) (forward), 5′-CATGATGTATAGATGCAGAGGTTTCTCA-3′ and (reverse) 5′-GTGCCTGGCTCTCTGTGA-3′; #3 (− 1400 ~ − 1600) (forward), 5′-AAAGCGGAGACTTGTCCAG-3′ and (reverse) 5′-AGGCTCCTCCCTCTTTCTGT-3′; #4 (− 1200 ~ − 1400) (forward), 5′-TCCGGGTAGGTTGGAGTGAGTA-3′ and (reverse) 5′-TCAACGTCTACTGAGCACTTAAGG-3′; #5 (− 1000 ~ − 1200) (forward), 5′-ACTGGACGGGCGC-3′ and (reverse) 5′-CCTGCCTCAGCCTCCG-3′; #6 (− 800 ~ − 1000) (forward), 5′-AGAATCGCTTGAACCAGGCA-3′ and (reverse) 5′-CTTGTTCTATCTGCATAAACCCGAG-3′; #7 (− 600 ~ − 800) (forward), 5′-ATAACCACATCCCACATGGGAG-3′ and (reverse) 5′-CACCTTCTCGCCTCCTTCG-3′; #8 (− 400 ~ − 600) (forward), 5′-CGCGCGCCCTTCTCA-3′ and (reverse) 5′-TTCCCCACACCGTCCTG-3′; #9 (− 200 ~ − 400) (forward), 5′-GGGTAAGAGGGCTCAGGG-3′ and (reverse) 5′-CGCGCGCGGAGG-3′; #10 (0 ~ − 200) (forward), 5′-GCCCTCTCCGGCTCAG-3′ and (reverse) 5′-GAGCGCGGGGCTG-3′; Axin2-TBE, (forward) 5′-TCTTGCCTTCCTCTCACTT-3′ and (reverse) 5′-GCTCATCTGAACCTCCTCTCT-3′; Axin2-ORF, (forward) 5′-AGGCCCGCTCGGATCTTTT-3′ and (reverse) 5′-GGGGAACATGGGGAGTCGTG-3′.

### Transmission electron microscopy (TEM)

PANC-1 cells were fixed in 2.5% glutaraldehyde without washing at 37 °C and further fixed with 2% osmium tetroxide buffer. Then, the fixed cells were dehydrated using a graded ethanol series and embedded in Epon. Electron microscopy was performed with a JEM-100CX-II TEM transmission electron microscope (Joel, Tokyo, Japan).

### Subcutaneous xenograft experiments

The subcutaneous xenograft mouse model was used to assess the tumor formation ability of PANC-1 cells stably expressing PVT1 treated with or without gemcitabine. All animal experiments were carried out in compliance with a protocol specifically approved for the use of laboratory animals by the Hubei University of Technology Animal Care and Use Committee. Four-week-old (18–22 g) male BALB/c nude mice were purchased from Vital River Laboratory Animal Technology (Beijing, China). PANC-1 cells (3 × 10^6^) stably expressing empty vector/PVT1 were resuspended in 100 μl phosphate-buffered saline (PBS) and subcutaneously injected into the left and right axillae of 3 female BALB/c nude mice per group. One week later, the animals were treated with gemcitabine at 50 mg/kg body weight via intraperitoneal injection twice a week. The length and width of the mouse tumors were measured every 5 days with calipers, and tumor volume (V) was calculated with the following formula: V = [(length × width × 2)/2]. Experimental mice were euthanized at the end of the observation period, and then the tumors were excised and weighed.

### PIK3C3/VPS34 kinase activity assay

PIK3C3/VPS34 kinase activity assay was performed as described previously [11]. Briefly, PIK3C3 was immunoprecipitated using an anti-ATG14 antibody after PVT1 or miR-619-5p overexpression with or without gemcitabine treatment. Then the immunoprecipitates were incubated with kinase reaction buffer (100 mM HEPES [ThermoFisher, 15,630,080] [pH 7.5], 300 mM NaCl, 2 mM CHAPS [ThermoFisher, 28,300], 10 mM MnCl2, 2 mM DTT, 100 μM ATP [Sangon Biotech, A600020]) and phosphatidylinositol substrate (Echelon, K-3000) and incubated at room temperature for 2 h. After the addition of the PtdIns3P detection buffer (provided by the kit, K-3004) and PtdIns3P detector protein (provided by the kit), the reaction mixture was then added to the PtdIns3P-coated microplate. The amount of PtdIns3P detector protein bound to the plate was determined through colorimetric detection of the absorbance at 450 nm. The concentration of PtdIns3P in the reaction mixture was calculated as the inverse of to the amount of PtdIns3P detector protein bound to the plate.

### Statistical analysis

All experiments were performed independently at least three times. All statistical analysis were performed using GraphPad Prism 6.0 software (GraphPad, La Jolla, CA, USA). All data are presented as the mean ± SD (standard deviation) from triplicates. Differences with a *p* value < 0.05 were statistically significant. Differences between two groups were analyzed by independent sample *t*-tests and differences among multiple groups by one-way ANOVA; *represents *P* < 0.05, **represents *P* < 0.01 and ***represents *P* < 0.001.

## Results

### PVT1 promotes pancreatic cancer cell resistance to gemcitabine in vitro and in vivo

**Fig. 1:**
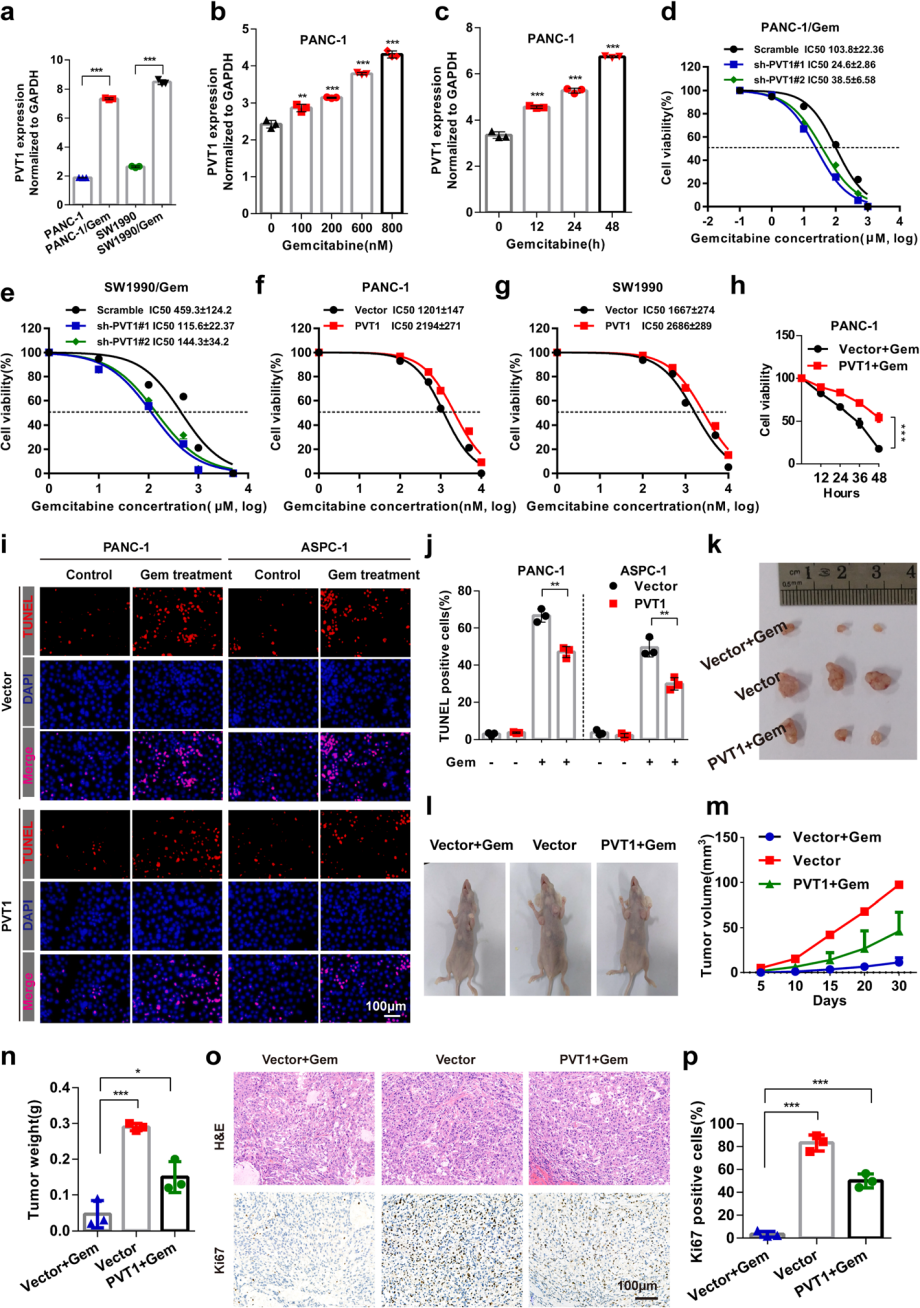
PVT1 promotes pancreatic cancer cell resistance to gemcitabine in vitro and in vivo*.***a** After nocodazole treatment for 12 h, the expression level of PVT1 in PANC-1 and SW1990 human pancreatic cancer cell lines and the PANC-1/Gem and SW1990/Gem gemcitabine resistant cell lines was examined by real-time qPCR. **b** and **c** PANC-1 cells were treated with gemcitabine at different concentrations and for different durations as indicated. Then, the expression levels of PVT1 were determined. **d-g** PANC-1/Gem and SW1990/Gem cells with stable PVT1knockdown and PANC-1 and SW1990 cells with stable PVT1 expression were treated with gemcitabine at different concentrations for 48 h, and cell viability was then measured by MTT assay. **h** PANC-1 cells stably expressing PVT1 were treated with gemcitabine (1 μM) for different durations as indicated, and cell viability was measured by MTT assay. **i** and **j** Apoptotic cells among PVT1 overexpressing PANC-1 and ASPC-1 cells treated with gemcitabine were analyzed by TUNEL assay, the number of TUNEL-positive cells was quantified. Scale bars: 100 μm. **k** and **l** Representative photographs of tumor-bearing mice in different groups and tumors excised from the mice were shown. **m** Growth curve showing changes in tumor volume in mice from different groups; growth was assessed every 5 days beginning from the injection and during gemcitabine (50 mg/kg) treatment. **n** Weight of the tumors excised from mice in each group. **o** and **p** Representative H&E staining images and immunohistochemical images of Ki67 in excised tumor tissues. Scale bars: 100 μm. Data were represented as mean ± SD, *P < 0.05; **P < 0.01; ***P < 0.001

To address the potential role of PVT1 in the gemcitabine sensitivity of pancreatic cancer cells, we first stably overexpressed PVT1 in the PANC-1 and SW1990 human pancreatic cancer cell lines and knocked down PVT1 expression in the gemcitabine (Gem) resistant PANC-1/Gem and SW1990/Gem cell lines by using lentiviral transfection. The expression of PVT1 was remarkably increased or decreased as expected (Additional file 1: Figure S1A and B). Interestingly, we found that the PVT1 level was higher in gemcitabine resistant cells than in the control group. We further compared the level of PVT1 between gemcitabine resistant and control cells after treating the cells with nocodazole, which was used to synchronize the cells at M phase, to avoid interference by differences in cell cycle stage. The results showed that PVT1 was significantly up-regulated (Fig. 1a). Furthermore, we found that gemcitabine treatment induced the up-regulation of PVT1 in both a dose- and time-dependent manner (Fig. 1b and c).

To determine the impact of these changes in PVT1 expression on gemcitabine sensitivity, cells were exposed with indicated concentrations of gemcitabine for 48 h. Cell viability assay showed that the depletion of PVT1 restored the sensitivity of PANC-1/Gem and SW1990/Gem cells to gemcitabine and decreased the gemcitabine IC50 value compared with that of the control cells (Fig. 1d and e). In contrast, PVT1 overexpression promoted gemcitabine resistance and increased the gemcitabine IC50 value in nromal PANC-1 and SW1990 cells (Fig. 1f and g). Moreover, we also confirmed that PVT1 overexpression increased the viability of PANC-1 and ASPC-1 cells under gemcitabine treatment in a time-dependent manner (Fig. 1h, Additional file 1: Figure S1C). We further investigated whether PVT1 affects the apoptosis rate of pancreatic cancer cells induced by gemcitabine. To that end, TUNEL assay was performed and the results showed that PVT1 overexpression significantly reduced the number of TUNEL-positive cells compared with that among the control cells (Fig. 1i and j). Additionally, compared with control cells, the caspase 3/7 activity was lower in PVT1 overexpressing cells but higher in cells transfected with PVT1 siRNA#1 (hereafter referred to as PVT1 siRNA) upon treatment with gemcitabine (Additional file 1: Figure S1D). The apoptosis rates of PVT1 overexpression and PVT1 knockdown cells were not significantly difference from those of the control groups in the absence of gemcitabine treatment (Fig. 1i and j, Additional file 1: Figure S1D).

To further assess the impact of PVT1 on pancreatic cancer sensitivity to gemcitabine in vivo, BALB/c nude mice bearing subcutaneous pancreatic xenograft tumors derived from PANC-1 cells stably expressing PVT1 were treated with 50 mg/kg gemcitabine. After 4 weeks of treatment, the tumors showed different responses to gemcitabine and the levels of PVT1 were increased in the excised tumors after gemcitabine treatment (Fig. 1k and l, Additional file 1: Figure S1E). The tumor volume and weight were decreased after gemcitabine treatment compared with those in the control group. However, the addition of PVT1 weakened the suppressive effect of gemcitabine on tumor growth (Fig. 1m and n). Moreover, PVT1 expression also contributed to the increased level of Ki67 in xenograft tumor tissues (Fig. 1o and p). Taken together, these data indicate that PVT1 was up-regulated under gemcitabine treatment conditions and that a high level of PVT1 promoted pancreatic cancer cell resistance to gemcitabine in vitro and in vivo.

### Gemcitabine resistance induced by PVT1 was associated with increased Wnt/β-catenin signaling pathway and autophagic activity

**Fig. 2:**
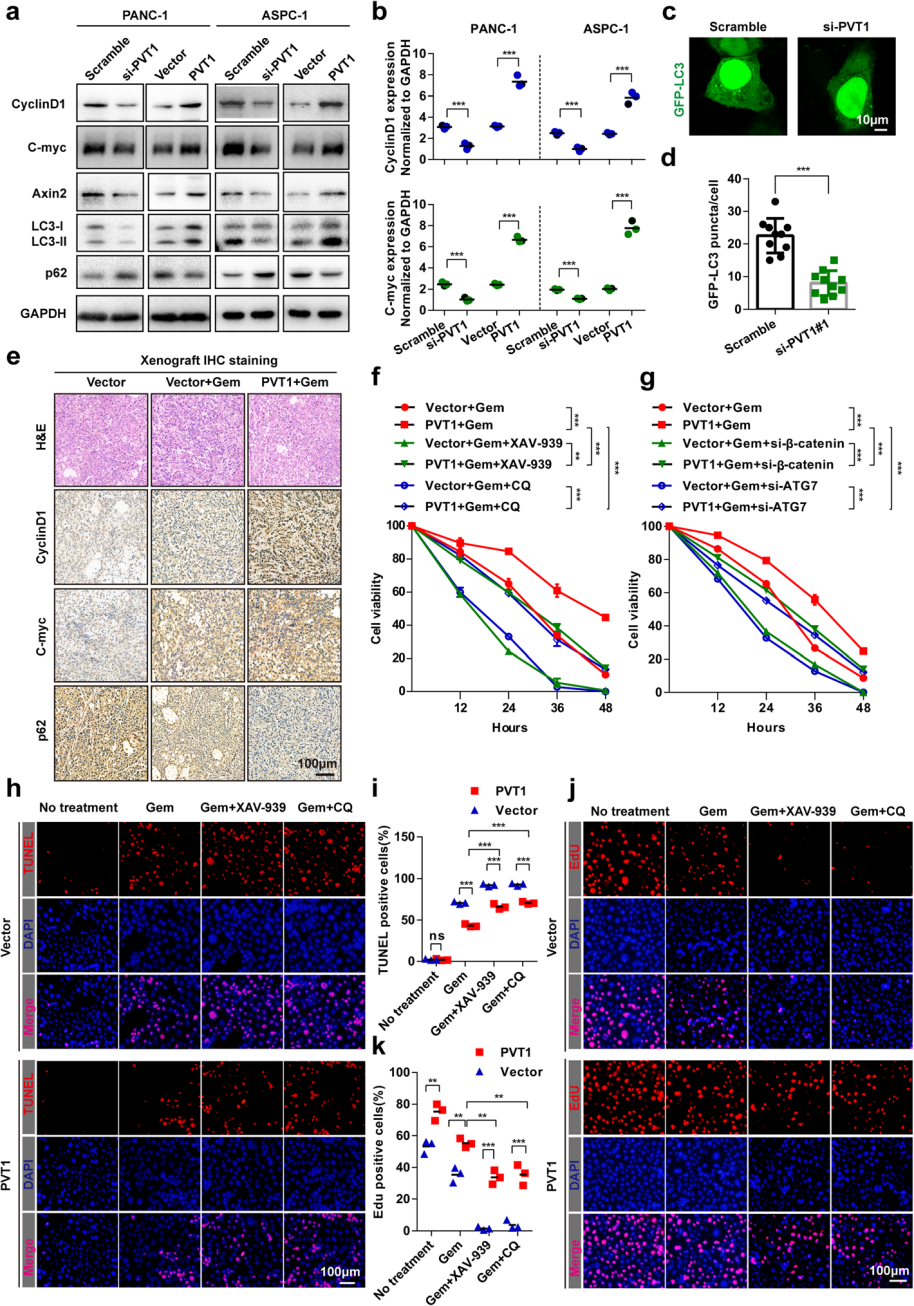
Gemcitabine resistance induced by PVT1 was associated with increased Wnt/β-catenin signaling pathway and autophagic activity. **a** Whole cell lysates from PANC-1 and ASPC-1 cells transfected with PVT1 overexpression plasmid or PVT1 siRNA were analyzed by western blotting using the indicated antibodies. **b** The expression levels of *CyclinD1* and *C-myc* in PANC-1 and ASPC-1 cells transfected with PVT1 overexpression plasmid or PVT1 siRNA were analyzed by real-time qRT-PCR. **c** and **d** Representative confocal images of GFP-LC3 puncta in PANC-1 cells transfected with PVT1 siRNA. The number of GFP-LC3 puncta was quantified using ImageJ software. (*n* = 10). Scale bars: 10 μm. **e** Representative H&E staining images and immunohistochemical images of CyclinD1, C-myc and p62 in excised xenograft tumor tissues. Scale bars: 100 μm. **f** PANC-1 cells stably expressing PVT1 were treated with gemcitabine (1 μM), XAV-939 (10 μM) or CQ (10 μM) for different durations as indicated, and cell viability was measured by MTT assay. **g** PANC-1 cells stably expressing PVT1 were transfected with β-catenin siRNA or ATG7 siRNA, and the cells were then treated with gemcitabine (1 μM) for different durations as indicated. Cell viability was measured by MTT assay. **h** and **i** Apoptotic cells among PVT1 overexpressed PANC-1 cells treated with gemcitabine (1 μM), XAV-939 (10 μM) or CQ (10 μM) were analyzed by TUNEL assay, and the number of TUNEL-positive cells was quantified. Scale bars: 100 μm. **j** and **k** Representative images and quantification of EdU incorporation in PVT1 overexpressing PANC-1 cells treated with gemcitabine (1 μM), XAV-939 (10 μM) or CQ (10 μM). Scale bars: 100 μm. Data were represented as mean ± SD, *P < 0.05; **P < 0.01; ***P < 0.001

Activation of both the Wnt/β-catenin signaling and autophagy pathways plays an essential role in chemoresistance in tumors, such as colon cancer and neuroblastoma [13, 17]. To explore whether the Wnt/β-catenin pathway and autophagy were involved in the gemcitabine resistance induced by PVT1 in human pancreatic cancer cells, we investigated the protein and mRNA expression levels of the Wnt/β-catenin target genes CyclinD1, C-myc and Axin2 after PVT1 overexpression or knockdown. The results indicated that at both the protein and mRNA levels, CyclinD1, C-myc and Axin2 were increased or decreased in PANC-1 and ASPC-1 cells transfected with PVT1 overexpression plasmid or PVT1 small interfering RNA, respectively (Fig. 2a and b, Additional file 1: Figure S2A). Moreover, we examined the effect of PVT1 depletion and overexpression on the levels of LC3 and p62/SQSTM1, the most commonly used autophagic flux markers of autophagosome formation [25]. We found that LC3-II levels were significantly reduced and that p62 levels were increased in PVT1 knockdown cells. In contrast, PVT1 overexpression increased LC3-II and decreased p62 levels (Fig. 2a). As GFP-LC3 puncta were also used to assess autophagosome formation, we carried out immunofluorescence experiments in PANC-1 cells and found that the number of GFP-LC3 puncta was significantly reduced after PVT1 knockdown (Fig. 2c and d). In addition, immunohistochemistry of tumors excised from the BALB/c nude mice showed that the expression levels of CyclinD1 and C-myc were much higher and that the level of p62 was lower in gemcitabine treated tumors than in untreated control tumors. Moreover, tumors stably expressing PVT1 showed the highest levels of CyclinD1 and C-myc and the lowest levels of p62 (Fig. 2e, Additional file 1: Figure S2B).

To confirm that Wnt/β-catenin signaling and autophagy protect against gemcitabine toxicity in pancreatic cancer cells, we further investigated whether Wnt/β-catenin signaling or autophagy inhibition would enhance the sensitivity of pancreatic cancer cells to gemcitabine using XAV-939 (a small-molecule inhibitor of Wnt/β-catenin signaling) and chloroquine (CQ, an autophagy inhibitor) or small interfering RNA targeting β-catenin and ATG7, which are critical proteins in Wnt/β-catenin signaling and autophagy, respectively. The sensitivity of PANC-1 cells to gemcitabine was significantly restored after XAV-939 or CQ treatment (Fig. 2f). Similar effects on gemcitabine sensitivity in PANC-1 cells were found in response to β-catenin siRNA or ATG7 siRNA transfection (Fig. 2g, Additional file 1: Figure S2C). Moreover, compared with normal conrols, cells stably expressing PVT1 showed much more resistance to gemcitabine treatment under Wnt/β-catenin signaling or autophagy inhibition conditions (Fig. 2f and g). Additionally, combined treatment with gemcitabine and XAV-939 or CQ significantly increased the number of TUNEL-positive cells among normal or PVT1 stably expressing PANC-1 cells. However, the apoptosis rate in PANC-1 cells stably expressing PVT1 was much lower than that in normal controls (Fig. 2h and i). Furthermore, an EdU incorporation assay was performed and showed that only approximately 1.85 ~ 6.88% of control cells incorporated EdU compared with approximately 28.55 ~ 41.52% of cells stably expressing PVT1 cells under gemcitabine treatment conditions, and XAV-939 or CQ treatment significantly inhibited the incorporation of EdU in PANC-1 cells. Taken together, these results demonstrated that PVT1 induced gemcitabine resistance is associated with increased Wnt/β-catenin signaling and autophagic activity, and inhibition of Wnt/β-catenin signaling or autophagy increased the sensitivity of human pancreatic cancer cells to gemcitabine.

### PVT1 promotes the Wnt/β-catenin signaling pathway through enhancing Pygo2 expression

**Fig. 3:**
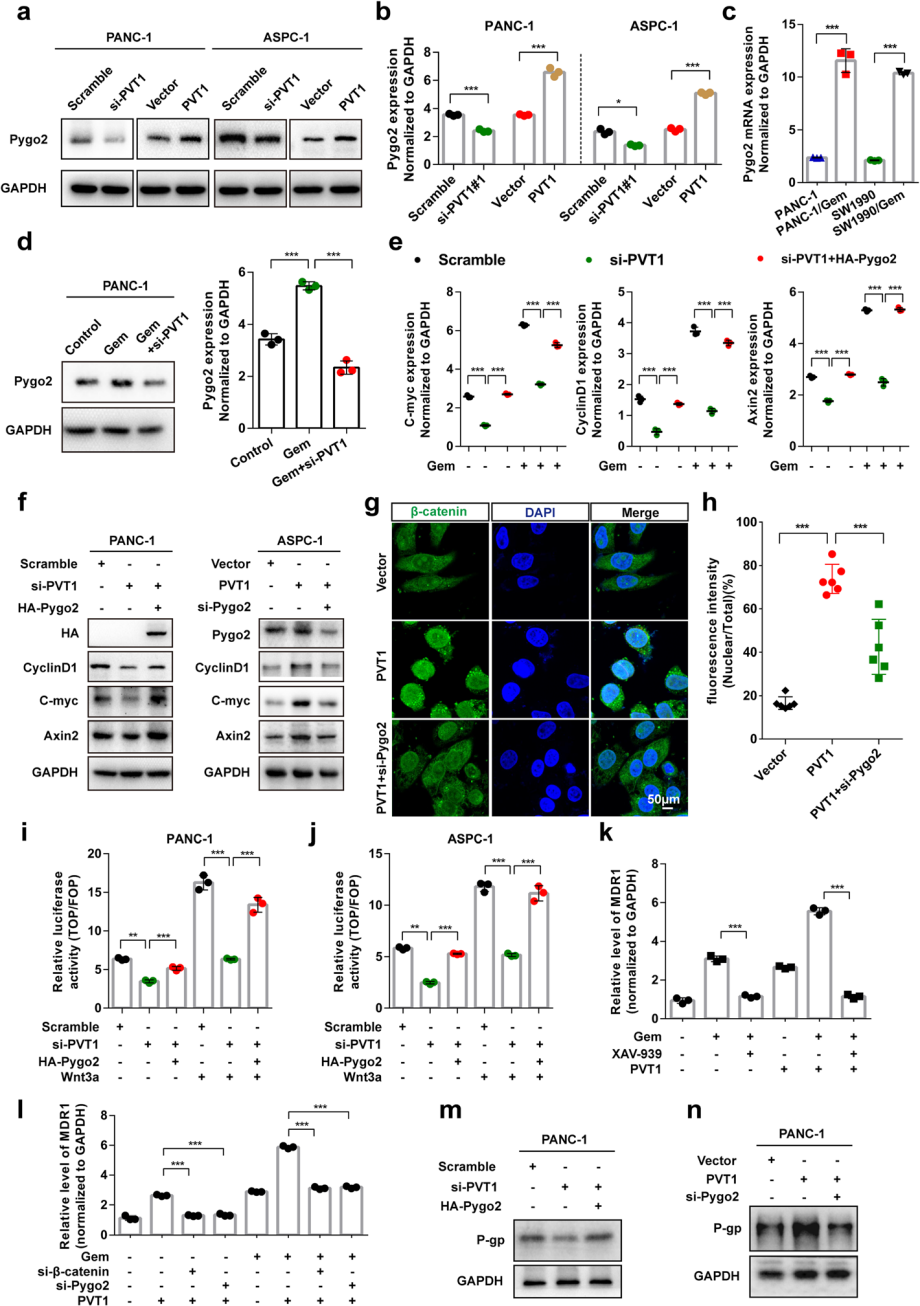
PVT1 promotes the Wnt/β-catenin signaling pathway through enhancing Pygo2 expression. **a** and **b** The protein and mRNA expression levels of Pygo2 in PANC-1 and ASPC-1 cells transfected with PVT1 overexpression plasmid or PVT1 siRNA were analyzed by western blotting and real-time qRT-PCR, respectively. **c** The mRNA expression level of Pygo2 in PANC-1, SW1990 and the gemcitabine resistant PANC-1/Gem, SW1990/Gem cell lines was examined by real-time qPCR. **d** The mRNA and protein expression levels of Pygo2 in PANC-1 cells transfected with PVT1 siRNA with or without gemcitabine treatment were analyzed. **e** The expression levels of *C-myc*, *CyclinD1* and *Axin2* in PANC-1 cells transfected with PVT1 siRNA or HA-Pygo2 plasmid treated with or without gemcitabine were analyzed by real-time qRT-PCR. **f** The whole cell lysates of PANC-1 and ASPC-1 cells transfected with PVT1 overexpression plasmid or PVT1 siRNA combined with Pygo2 overexpression plasmid or Pygo2 siRNA transfection were analyzed by western blotting using the indicated antibodies. **g** and **h** The cytoplasmic and nuclear distributions of β-catenin after PVT1 or Pygo2 siRNA transfection were assessed by immunofluorescence. The ratio of nuclear fluorescence intensity/total fluorescence intensity was quantified using GraphPad Prism 6.0 software. Scale bars: 50 μm. **i** and **j** TCF transcriptional activity was compared between PVT1 knockdown and Pygo2 overexpressing cells with or without Wnt3a (100 ng/ml). Cells were transiently transfected with TOP/FOP Flash reporter, and the luciferase activity was measured. **k-n** The protein and mRNA levels of MDR1 (P-gp) were measured in PANC-1 cells with PVT1 overexpression or knockdown with or without Pygo2/β-catenin knockdown treated with gemcitabine (1 μM) or XAV-939 (10 μM). Data were represented as mean ± SD, *P < 0.05; **P < 0.01; ***P < 0.001

Pygopus2 (Pygo2), a newly identified components of the Wnt/β-catenin signaling pathway, was reported to activate MDR1 expression and mediate chemoresistance in human brain glioma and breast cancer via the Wnt/β-catenin signaling pathway by our team and other researchers [19, 26]. To investigate the mechanism of PVT1 in pancreatic cancer gemcitabine resistance, the regulation of PVT1 and Pygo2 expression was assessed, the results of which indicated that PVT1 overexpression significantly up-regulated both the protein and mRNA levels of Pygo2. In contrast, PVT1 knockdown significantly down-regulated Pygo2 protein and mRNA expression (Fig. 3a and b). We also found that the mRNA expression of Pygo2 was much higher in gemcitabine resistant pancreatic cancer cell lines than in control cells (Fig. 3c). Moreover, we found that both the protein and mRNA levels of Pygo2 were increased after gemcitabine treatment and decreased after combined treatment with PVT1 siRNA (Fig. 3d).

To investigate whether PVT1 regulates the Wnt/β-catenin signaling pathway through enhancing Pygo2, we first examined the expression of the Wnt/β-catenin signaling target genes C-myc, CyclinD1 and Axin2. The results showed that Pygo2 overexpression restored the inhibitory effect of PVT1 knockdown on the mRNA and protein levels of C-myc, CyclinD1 and Axin2 with or without gemcitabine treatment (Fig. 3e and f). Immunofluorescence analysis of β-catenin revealed that the β-catenin nuclear fluorescence signal was significantly increased following the overexpression of PVT1, but restored by the depletion of Pygo2 (Fig. 3g and h). Consistent with this result, TOPFlash assays revealed much lower TCF/LEF transcriptional activity in PVT1 siRNA transfected PANC-1 and ASPC-1 cells both with or without Wnt3a treatment. Moreover, Pygo2 overexpression restored the decrease in TCF/LEF transcriptional activity induced by PVT1 knockdown (Fig. 3i and j). One of the mechanisms by which Wnt/β-catenin signaling induces chemoresistance is by up-regulating the expression of P-glycoprotein (P-gp/ABCB1, encoded by the *MDR1* gene), increasing the energy-dependent efflux of cytotoxic drugs from cancer cells [27]. We then assessed the effects of PVT1 on the expression of *MDR1*, the results of which indicated that PVT1 specifically increased the mRNA level of *MDR1*. Moreover, the increase in *MDR1* mRNA was inhibited by either treatment with XAV-939 or depletion of β-catenin and Pygo2 (Fig. 3k and l). Similar effects on the regulatory effect of PVT1 on the P-gp protein levels were observed (Fig. 3m and n). These data indicated that PVT1 promotes the Wnt/β-catenin signaling pathway through enhancing Pygo2 expression.

### Up-regulation of Pygo2 elevates PVT1 levels through the Wnt/β-catenin signaling pathway

**Fig. 4:**
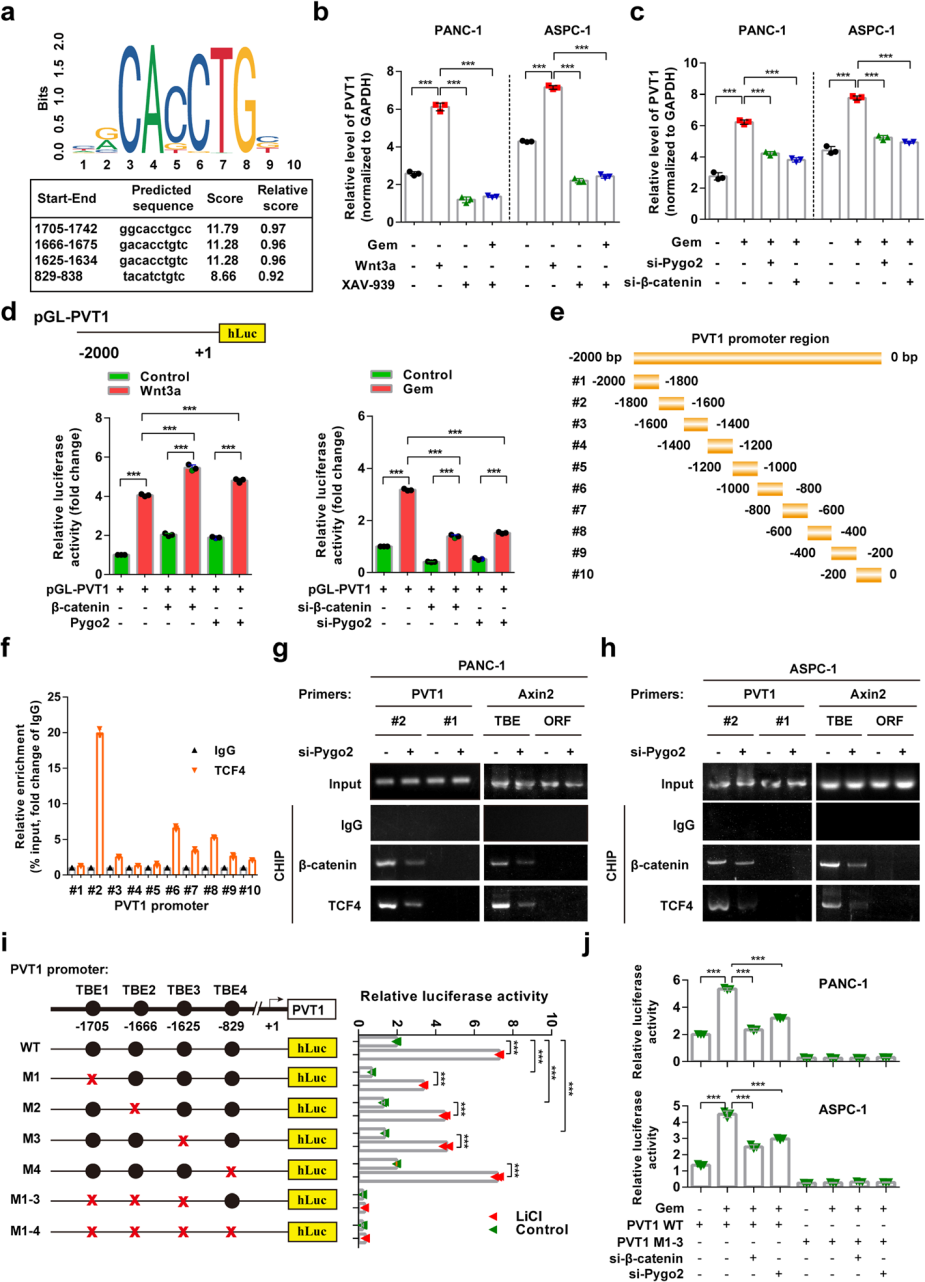
Up-regulation of Pygo2 elevated PVT1 levels through the Wnt/β-catenin signaling pathway. **a** TCF4 binding sequence in the PVT1 promoter region predicted with the JASPAR website; the predicted sequence and score were shown. **b** Relative levels of PVT1 in PANC-1 and ASPC-1 cells treated with the Wnt/β-catenin signaling activator Wnt3a (100 ng/ml) and inhibitor XAV939 (10 μM). **c** Relative levels of PVT1 in PANC-1 and ASPC-1 cells treated with gemcitabine (1 μM) with or without Pygo2 siRNA or β-catenin siRNA transfection. **d** pGL-PVT1 promoter reporter luciferase activity in PANC-1 cells transfected with β-catenin and Pygo2 overexpression vector or Pygo2 siRNA and β-catenin siRNA treated with Wnt3a (100 ng/ml) or gemcitabine (1 μM). **e** Deletion mutants of the PVT1 promoter. **f** PANC-1 cells were subjected to ChIP assay using an antibody against TCF4, and enrichment of the PVT1 promoter fragments was shown. **g** and **h** ChIP assay in PANC-1 and ASPC-1 cells with the indicated primers and antibodies with or without Pgyo2 siRNA transfection. The Axin2 promoter was used as a positive control, and the primers to amplify its ORF regions were used as negative controls. **i** Schematic diagram of the generated luciferase reporter plasmids containing wild-type and mutant predicted TCF4 binding elements and relative luciferase activity after the transfection of HEK293T cells with these plasmids with or without LiCl (50 mM) treatment. **j** The effects of β-catenin and Pygo2 knockdown on the luciferase activity in PANC-1 and ASPC-1 cells transfected with wild-type PVT1 and PVT1 1–3 deletion mutant reporter plasmids with or without gemcitabine treatment. Data were represented as mean ± SD, *P < 0.05; **P < 0.01; ***P < 0.001

Through sequence alignment using the JASPAR website (http://jaspar.genereg.net/analysis) [28], we were surprised to find four probable TCF4 binding regions (TBE1–4) in a 2-kb stretch of the 5′-promoter region of PVT1 (Fig. 4a). To investigate the presence of a positive feedback loop between Wnt/β-catenin signaling and PVT1 expression, we evaluated PVT1 expression when Wnt/β-catenin signaling was impaired or activated. The activation of β-catenin by Wnt3a enhanced the expression of PVT1, while inhibition of β-catenin by XAV-939 reduced the expression of PVT1 both with and without gemcitabine treatment. Moreover, the knockdown of either Pygo2 or β-catenin decreased PVT1 expression (Fig. 4b and c). By linking the PVT1 promoter region (− 2000 ~ + 1 bp) to a luciferase reporter, we found that the overexpression of either β-catenin or Pygo2 up-regulated PVT1 reporter activity under Wnt3a or gemcitabine treatment, while β-catenin or Pygo2 knockdown decreased PVT1 reporter activity under Wnt3a or gemcitabine treatment (Fig. 4d, Additional file 1: Figure S3A and B). To further investigate the regulatory effect of Wnt/β-catenin on PVT1 transcription, we performed chromatin immunoprecipitation (ChIP) assays to assess the potential TCF4/β-catenin binding element in the PVT1 promoter. The results revealed that the − 1600 ~ − 1800 promoter region (#1 TBE region) before the transcription start site (TSS) was most abundant among regions enriched by the TCF4 antibody (Fig. 4e and f). Moreover, Pygo2 knockdown significantly decreased enrichment of the PVT1 promoter − 1600 ~ − 1800 region, and enriched Axin2 TBE and ORF regions were used as positive and negative controls, respectively (Fig. 4g and h). Furthermore, a series of luciferase reporter plasmids carrying the wild-type or mutant PVT1 promoter region (TBE1–4) were generated. As shown in Fig. 4i, after the transfection of HEK293T cells with the luciferase reporter plasmids, we found that TBE1, TBE2 and TBE3 deletion significantly reduced luciferase activity, whereas no effect on luciferase activity was observed after TBE4 deletion. The deletion of both TBE1–3 and TBE1–4 completely abolished luciferase activity with or without the activation of β-catenin by inhibition of GSK3β using LiCl. Additionally, knockdown of Pygo2 or β-Catenin expression in PANC-1 and ASPC-1 cells significantly reduced luciferase activity driven by the wild-type fragment, but not the TBE-mutated fragment (Fig. 4j). These findings indicate that Pygo2 mediated Wnt/β-catenin signaling activates PVT1 expression via direct binding to the PVT1 promoter.

### PVT1 directly targets miR-619-5p

**Fig. 5:**
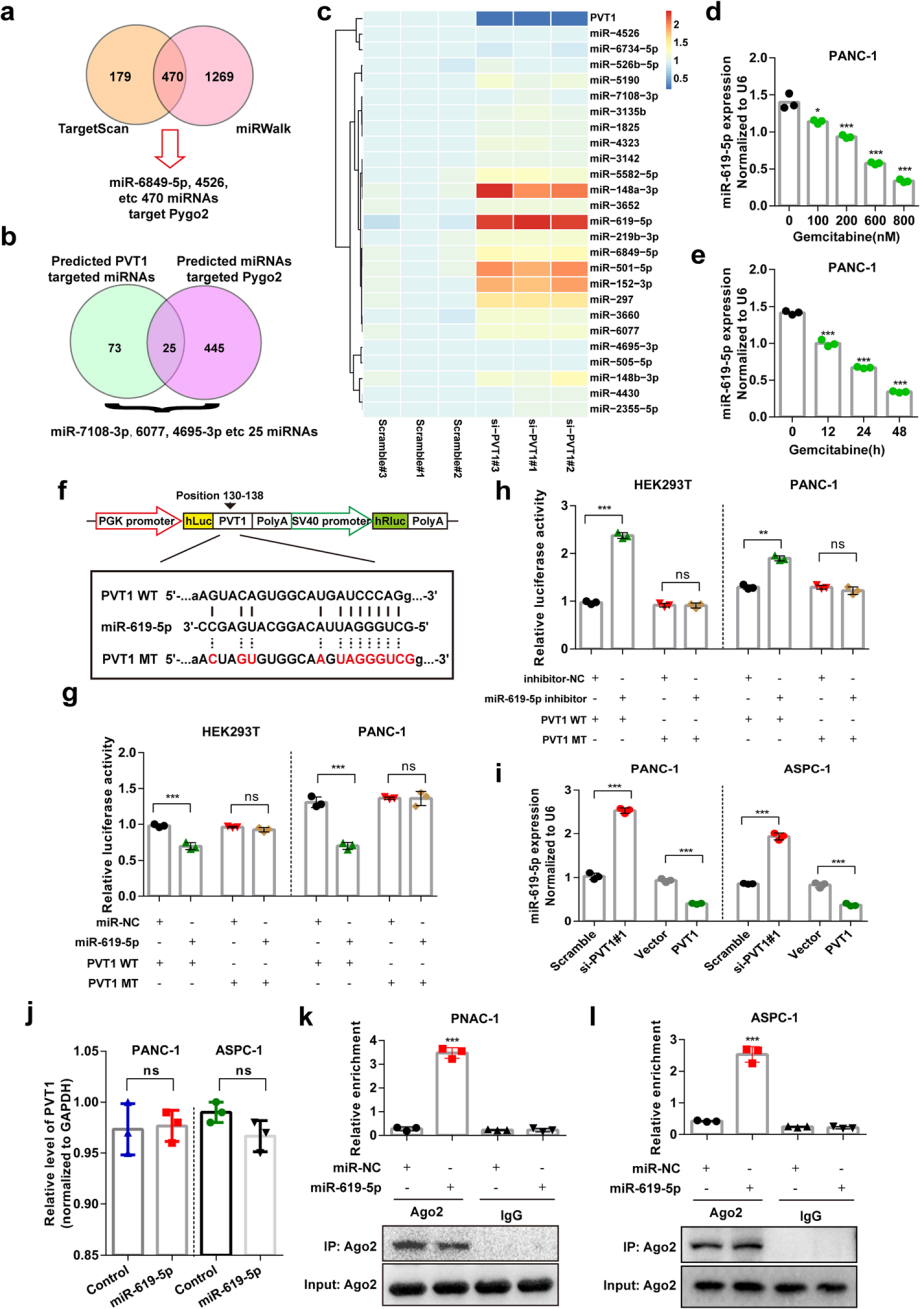
PVT1 directly targets miR-619-5p. **a** and **b** Potential miRNAs that target both Pygo2 and PVT1 were predicted using the miRWalk and TargetScan databases. **c** The expression of predicted miRNAs that target both Pygo2 and PVT1 was assessed by qRT-PCR after PVT1 transfected with siRNA for 48 h. Heatmap were drawn by using ImageGP online software (http://www.ehbio.com/ImageGP/). **d** and **e** The expression of miR-619-5p was measured in PANC-1 cells treated with indicated concentration and time. **f** The predicted miR-619-5p binding sequence in PVT1 and the generation of dual-luciferase reporter plasmids of wild-type (WT) or mutant (MT) were shown. **g** Luciferase activity assays were performed in HEK293T or PANC-1 cells co-transfected with PVT1 WT or PVT1 MT and miR-NC or miR-619-5p. **h** Luciferase activity assays were performed in HEK293T or PANC-1 cells co-transfected with PVT1 WT or PVT1 MT and miR-619-5p inhibitor or inhibitor-NC. **i** The relative expression of miR-619-5p in PANC-1 and ASPC-1 cells transfected with PVT1 siRNA or PVT1 overexpression plasmid. **j** The relative expression of PVT1 in PANC-1 and ASPC-1 cells transfected with miR-619-5p mimics. **k** and **l** Anti-Ago2 RIP was performed in PANC-1 and ASPC-1 cells transfected with miR-619-5p mimics or miR-NC, followed by qRT-PCR to detect PVT1 enrichment. Data were represented as mean ± SD, *P < 0.05; **P < 0.01; ***P < 0.001

One of the mechanisms by which lncRNAs function in cell regulation is competitively adsorbing miRNA as a ceRNA, which results in the loss or reduction of miRNA function and controls the regulatory effect of miRNA on target genes [4, 29]. By fractionated nuclear and cytoplasmic RNA analysis in PANC-1 and ASPC-1 pancreatic cancer cells, we found that PVT1 was distributed in both the cytoplasm and nucleus (Additional file 1: Figure S4A). We then asked whether PVT1 up-regulates Pygo2 expression through adsorbing one such miRNA. Bioinformatic analysis with several online bioinformatics databases predicted approximately 25 miRNAs that target both PVT1 and Pygo2 (Fig. 5a and b). Expression of the predicted miRNAs was screened in PANC-1 cells after PVT1 knockdown. The results showed that several miRNAs were up-regulated after PVT1 knockdown, and miR-619-5p displayed the most significant increase (Fig. 5c). We also found that the level of miR-619-5p was decreased after gemcitabine treatment in both a concentration- and time-dependent manner (Fig. 5d and e). To investigate whether PVT1 directly binds and regulates miR-619-5p, we performed a luciferase activity assay using a luciferase expression vector containing the PVT1 segment containing the putative miR-619-5p binding sites (Fig. 5f). The results showed that the activity of the luciferase reporter vector carrying the PVT1 WT sequence was significantly decreased by miR-619-5p mimics compared with that in the control groups, but mutation of the putative miR-619-5p binding sites completely eliminated these inhibitory effects in HEK293T and PANC-1 cells (Fig. 5g). In contrast, the luciferase assay showed that miR-619-5p inhibitor had the opposite effect (Fig. 5h). The expression of miR-619-5p was further confirmed in both PANC-1 and ASPC-1 cells transfected with PVT1 siRNA and overexpression plasmids (Fig. 5i); however, miR-619-5p up-regulation failed to alter PVT1 expression (Fig. 5j). miRNAs repress translation and degrade mRNA in an Ago2-dependent manner by binding their targets. We then performed anti-Ago2 RNA immunoprecipitation (RIP) in PANC-1 and ASPC-1 cells transiently overexpressing miR-619-5p to pull down PVT1 using anti-Ago2 antibodies or control IgG and analyzed the immunoprecipitates by qPCR. The amount of PVT1 pulled down with anti-Ago2 antibodies was significantly increased in cells transfected with miR-619-5p mimics compared to control cells (Fig. 5k and l). Collectively, these data demonstrated that PVT1 directly targets miR-619-5p.

### miR-619-5p negatively regulates Pygo2 and ATG14 expression

**Fig. 6:**
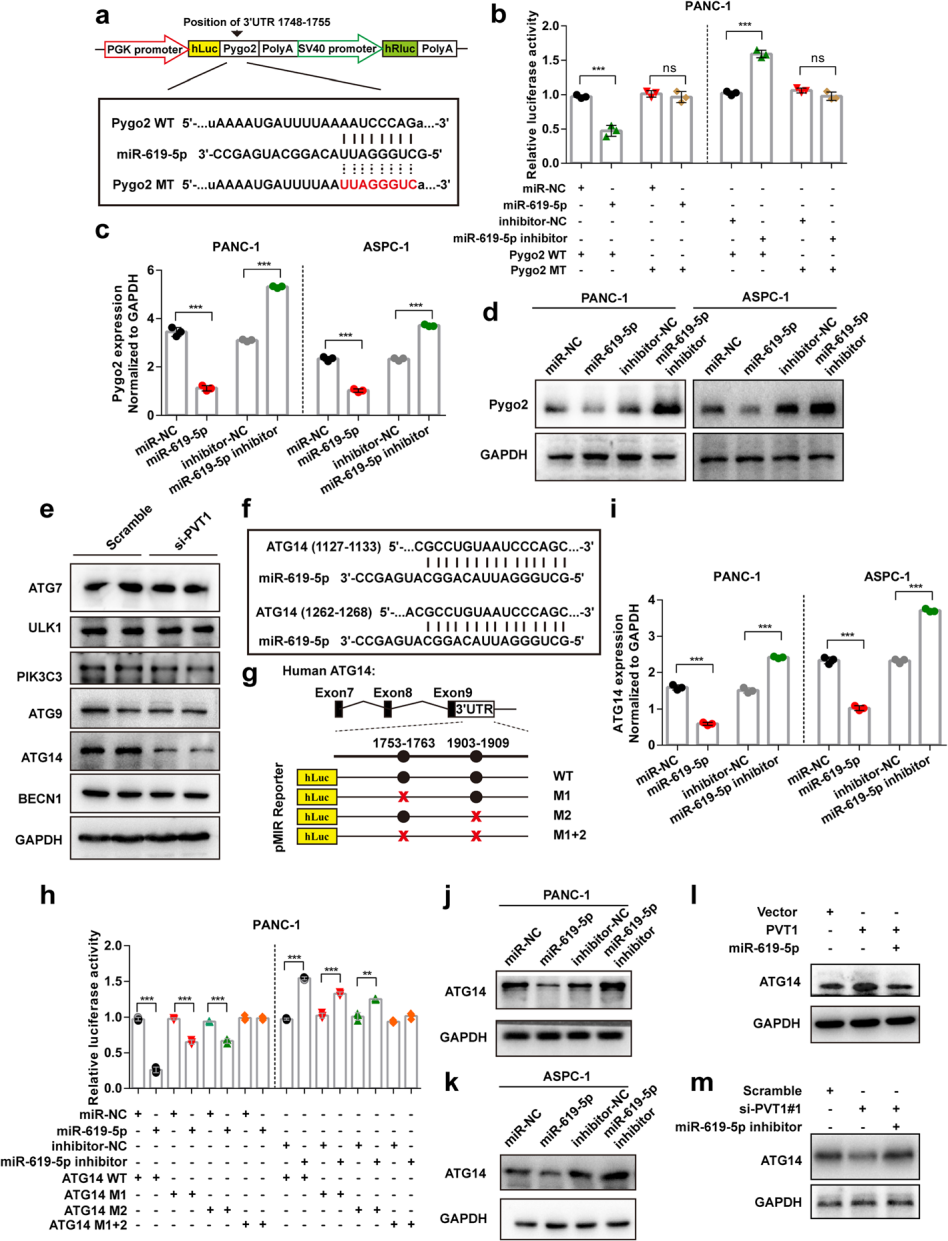
miR-619-5p negatively regulates Pygo2 and ATG14 expression. **a** The predicted miR-619-5p binding sequence in the Pygo2 3’UTR and the generation of dual-luciferase reporter plasmids of wild-type (WT) or mutant (MT) were shown. **b** Luciferase activity assays were performed in PANC-1 cells co-transfected with Pygo2 WT or Pygo2 MT and miR-619-5p mimic or miR-619-5p inhibitor. **c** and **d** The mRNA and protein levels of Pygo2 in PANC-1 and ASPC-1 cells after transfection with miR-619-5p mimics or miR-619-5p inhibitor. **e** Western blotting in PANC-1 cells transfected with PVT1 siRNA was carried out using the indicated antibodies. **f** and **g** The miR-619-5p binding sequence in the ATG14 3’UTR and the generation of dual-luciferase reporter plasmids of wild-type (WT) or mutant (MT) were shown. **h** Luciferase activity assays were performed in PANC-1 cells co-transfected with ATG14 WT or ATG14 MT and miR-619-5p mimic or miR-619-5p inhibitor. **i-k** The mRNA and protein levels of ATG14 in PANC-1 and ASPC-1 cells after transfection with miR-619-5p mimics or miR-619-5p inhibitor. **l** and **m** The expression of ATG14 after co-transfection with PVT1 and miR-619-5p mimics or PVT1 siRNA and miR-619-5p inhibitor. Data were represented as mean ± SD, *P < 0.05; **P < 0.01; ***P < 0.001

To further determine whether miR-619-5p can down-regulate Pygo2 expression, we performed dual-luciferase reporter assays in PANC-1 cells transfected with a luciferase plasmid harboring the predicted miR-619-5p binding sequence in the 3’UTR of Pygo2 and miR-619-5p mimics, miR-619-5p inhibitor or control sequences. The results revealed that miR-619-5p significantly inhibited luciferase activity driven by the luciferase reporter containing the WT Pygo2 3’UTR sequence but not the reporter containing the MT Pygo2 3’UTR sequence, but the miR-619-5p inhibitor had the observed opposite effect on luciferase activity (Fig. 6a and b). Moreover, the mRNA and protein levels of Pygo2 were determined after miR-619-5p overexpression or knockdown. The results revealed that miR-619-5p significantly decreased, while the miR-619-5p inhibitor significantly increased both the mRNA and protein levels of Pygo2 in PANC-1 and ASPC-1 cells (Fig. 6c and d).

As PVT1 promotes human pancreatic cancer gemcitabine resistance, which is also associated with cellular autophagic activity, we then investigated whether the PVT1/miR-619-5p axis could target autophagy-related gene(s). We first analyzed the expression of some autophagy-related genes after PVT1 knockdown, the results of which showed that the expression of ATG14 was significantly decreased (Fig. 6e). Moreover, we predicted potential miR-619-5p binding sequence(s) in the ATG14 3’UTR using TargetScan and found that the ATG14 3’UTR likely binds miR-619-5p (Fig. 6f). Furthermore, luciferase reporter assays showed that luciferase expression driven by WT ATG14 was significantly inhibited by co-transfection with the miR-619-5p mimic compared with that in the control PANC-1 and ASPC-1 cells. However, this inhibitory effect was abolished by mutation of the two putative miR-619-5p binding sites in the ATG14 3′ UTR. Cells co-transfection with miR-619-5p inhibitor showed the opposite effects (Fig. 6g and h). The mRNA and protein levels of ATG14 were further determined by qRT-PCR and western blotting, respectively, in PANC-1 and ASPC-1 cells and the results revealed that miR-619-5p mimics decreased ATG14 mRNA and protein expression, while miR-619-5p inhibitor increased PVT1 mRNA and protein expression (Fig. 6i-k). Additionally, we performed rescue assays to evaluate whether PVT1 regulates ATG14 by competing for miR-619-5p. The results showed that overexpression of PVT1 increased ATG14 protein levels and ectopic expression of miR-619-5p repressed this increase, but knockdown of PVT1 decreased ATG14 levels, and the miR-619-5p inhibitor restored this down-regulation (Fig. 6l and m). Collectively, these findings demonstrated that PVT1 up-regulates the expression of both Pygo2 and ATG14 by sponging miR-619-5p.

### The PVT1/miR-619-5p axis promotes autophagic activity by regulating ATG14

**Fig. 7:**
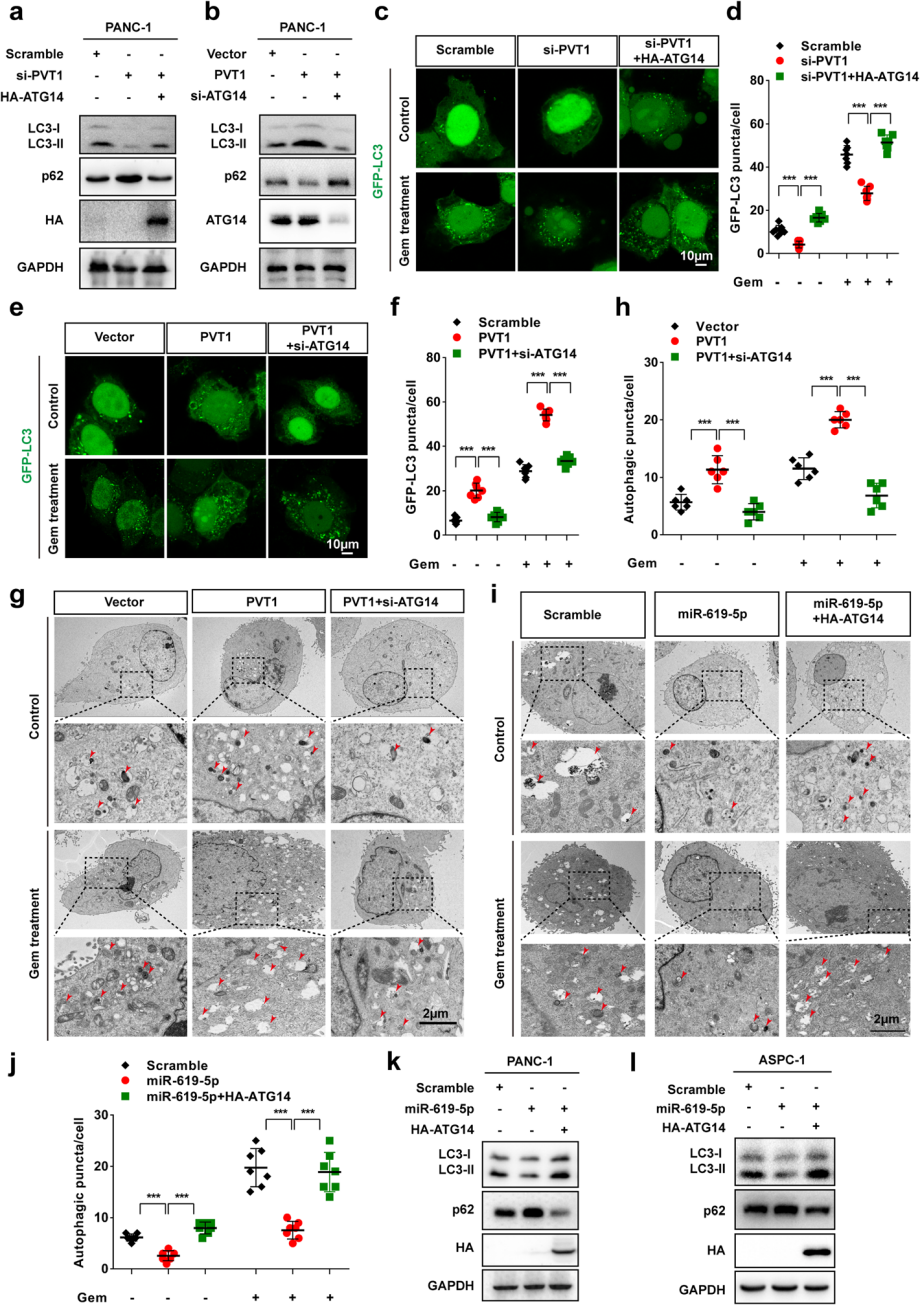
PVT1/miR-619-5p axis promotes autophagic activity by regulating ATG14. **a** Western blotting analysis of PANC-1 and ASPC-1 cells after PVT1 knockdown with or without ATG14 overexpression was carried out with the indicated antibodies. **b** Western blotting analysis of PANC-1 and ASPC-1 cells after PVT1 overexpression with or without ATG14 knockdown was carried out with the indicated antibodies. **c** and **d** Representative confocal images of GFP-LC3 puncta in PANC-1 cells transfected with PVT1 siRNA with or without co-transfection of ATG14 overexpression plasmid with and without gemcitabine treatment. The number of GFP-LC3 puncta was quantified using ImageJ software. (*n* = 10). Scale bars: 10 μm. **e** and **f** Representative confocal images of GFP-LC3 puncta in PANC-1 cells transfected with PVT1 overexpression plasmid with or without co-transfection with ATG14 siRNA with and without gemcitabine treatment. The number of GFP-LC3 puncta was quantified using ImageJ software. (n = 10). Scale bars: 10 μm. **g** and **h** Representative electronic micrographs of the autophagosomes or autolysosomes of PANC-1 cells co-transfected with PVT1 overexpression plasmid and/or ATG14 siRNA with or without gemcitabine treatment. Red arrows indicate autophagic structures. The number of autophagic structures per cell was quantified (n = 10). Scale bars: 2 μm. **i** and **j** Representative electronic micrographs of the autophagosomes or autolysosomes of PANC-1 cells co-transfected with miR-619-5p mimics and/or ATG14 siRNA with or without gemcitabine treatment. Red arrows indicate autophagic structures. The number of autophagic structures per cell was quantified (n = 10). Scale bars: 2 μm. **k** and **l** Western blotting analysis of PANC-1 and ASPC-1 cells after transfection with miR-619-5p mimics with or without ATG14 overexpression was carried out with the indicated antibodies. Data were represented as mean ± SD, *P < 0.05; **P < 0.01; ***P < 0.001

To investigate whether PVT1 promotes autophagic activity through ATG14, we performed several rescue assays. We found that PVT1 knockdown decreased LC3-II levels and increased p62 levels, whereas ectopic expression of HA-ATG14 reversed these alterations. In contrast, overexpression of PVT1 increased LC3-II levels and decreased p62 levels, and ATG14 depletion restored these effects in PANC-1 cells (Fig. 7a and b). Moreover, we carried out immunofluorescence experiments to access GFP-LC3 puncta in PANC-1 cells and found that the number of GFP-LC3 puncta was significantly reduced after PVT1 knockdown, with this change rescued after HA-ATG14 co-expression, whereas the number of GFP-LC3 puncta was markedly increased after PVT1 overexpression and restored by ATG14 knockdown with or without gemcitabine treatment (Fig. 7c-f). To confirm our hypothesis, we further detected the effect of PVT1 overexpression and ATG14 knockdown on autophagic vacuoles by using transmission electron microscopy. We observed a significant increase in autophagic vacuoles in PVT1 overexpressing PANC-1 cells and a restored phenotype in ATG14 siRNA co-transfected PANC-1 cells both with or without gemcitabine treatment (Fig. 7g and h). Additionally, we found that miR-619-5p mimics significantly decreased the number of autophagic vacuoles in PANC-1 cells both with or without gemcitabine treatment. Moreover, expression of miR-619-5p mimics and HA-ATG14 overexpression restored the number of autophagic vacuoles (Fig. 7i and j). Furthermore, western blotting revealed that miR-619-5p significantly reduced LC3-II levels and increased p62 levels, whereas ATG14 overexpression restored the levels of both LC3-II and p62 (Fig. 7k and l). These results indicated that the PVT1/miR-619-5p axis promotes autophagic activity by regulating ATG14.

### PVT1 interacts with ATG14 and promotes PtdIns3K-C1 complex assembly

**Fig. 8:**
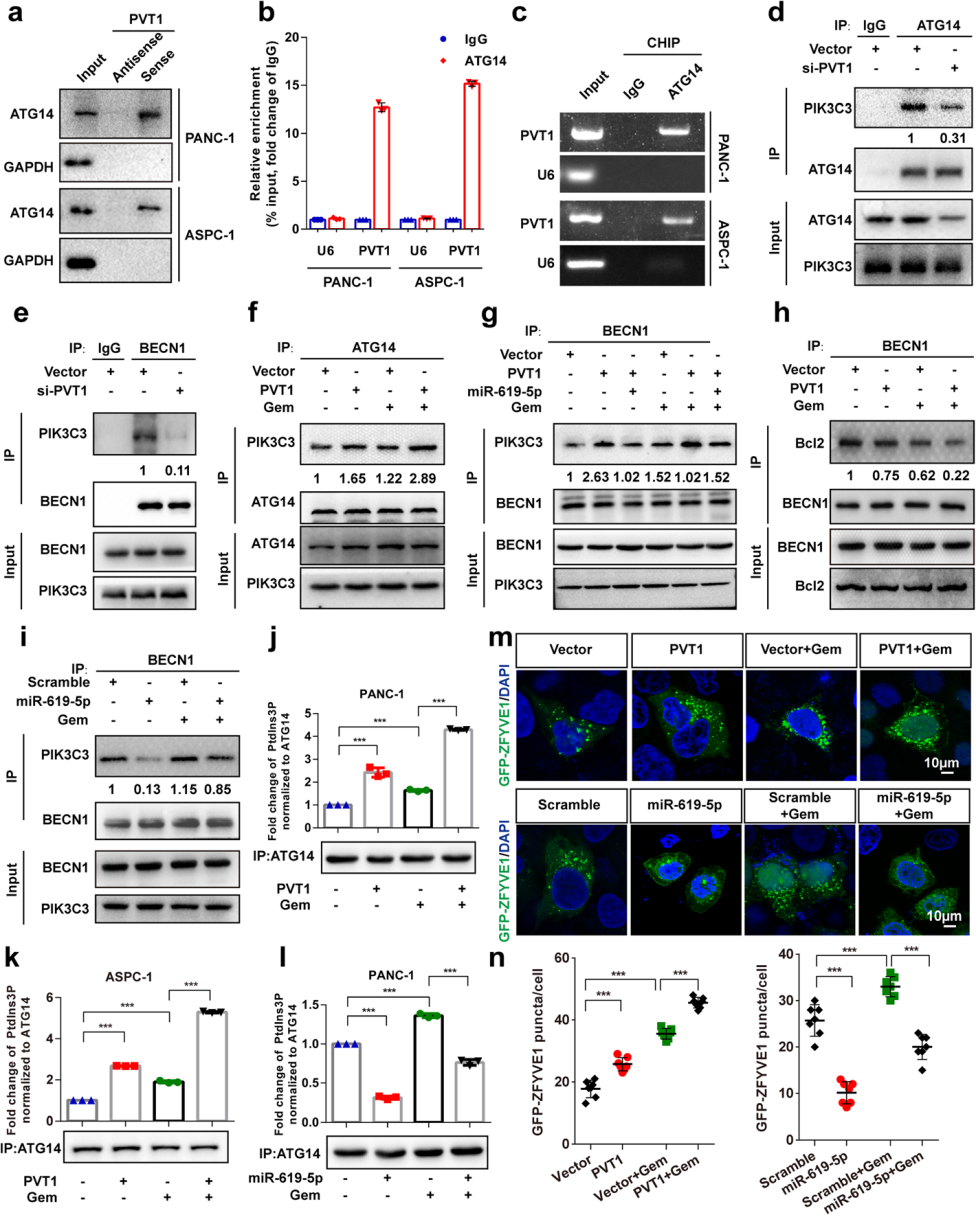
PVT1 interacts with ATG14 and promotes PtdIns3K-C1 complex assembly. **a** The interaction between PVT1 and ATG14 in PANC-1 and ASPC-1 cells was confirmed by RNA pulldown followed by western blotting. **b** and **c** qRT-PCR analysis of PVT1 following RNA immunoprecipitation (RIP) assays in PANC-1 and ASPC-1 cells using anti-ATG14 antibody. RNA enrichment was determined relative to the IgG control. U6 was used as a non-specific control. **d** and **e** The interaction between PIK3C3 and ATG14 or BECN1 after PVT1 knockdown in PANC-1 cells. Immunoprecipitated endogenous PIK3C3 was quantified using Image Lab software and normalized against the amount of PIK3C3 in whole-cell lysates. **f** and **g** The interaction between PIK3C3 and ATG14 or BECN1 after PVT1 overexpression and/or miR-619-5p co-transfection in PANC-1 cells with or without gemcitabine (1 μM) treatment. Immunoprecipitated endogenous PIK3C3 was quantified using Image Lab software and normalized against the amount of PIK3C3 in whole-cell lysates. **h** The interaction between Bcl2 and BECN1 after PVT1 overexpression in PANC-1 cells with or without gemcitabine (1 μM) treatment. Immunoprecipitated endogenous Bcl2 was quantified using Image Lab software and normalized against the amount of PIK3C3 in whole-cell lysates. **i** The interaction between PIK3C3 and BECN1 after the overexpression of miR-619-5p mimics in PANC-1 cells with or without gemcitabine (1 μM) treatment. Immunoprecipitated endogenous PIK3C3 was quantified using Image Lab software and normalized against the amount of PIK3C3 in whole-cell lysates. **j-l** Different PtdIns3K-C1 complex components were immunoprecipitated from PANC-1 and ASPC-1 cells overexpressing PVT1 or miR-619-5p mimics with or without gemcitabine (1 μM) treatment with ATG14 antibody. PIK3C3 activity was measured by analyzing PtdIns3P production using ELISA as described in the Materials and Methods section. The fold change in PtdIns3P activity was calculated based on the concentration of PtdIns3P and normalized to the amount of ATG14 used in the assay. **m** and **n** Representative confocal images of GFP-ZFYVE1 puncta in control or PVT1- or miR-619-5p-transfected PANC-1 cells with or without gemcitabine induction. The numbers of GFP-ZFYVE1 puncta was quantified (*n* = 10). Scale bars: 10 μm. Data were represented as mean ± SD, *P < 0.05; **P < 0.01; ***P < 0.001

LncRNAs have been reported to exert their biological functions by interacting with certain proteins [30]. Interestingly, PVT1 was predicted to bind ATG14 using the online alignment tool RPISeq (http://pridb.gdcb.iastate.edu//RPISeq/) [31]. To confirm this finding, RNA pull-down assays with biotin-labeled PVT1 sense and antisense controls were perfomed in PANC-1 and ASPC-1 cells. The results indicated the presence of ATG14 among the PVT1 sense pull-down components (Fig. 8a). Moreover, we performed an RNA immunoprecipitation (RIP) assay and found that PVT1 was significantly enriched by ATG14 antibody compared with IgG (Fig. 8b and c). Based on the interaction of PVT1 with ATG14 and its stimulatory effect on autophagic processes, we examined whether PVT1 affectes assembly of the PtdIns3K-C1 complex. The results indicated that PVT1 knockdown decreased the interaction of endogenous PIK3C3 with BECN1 and ATG14 (Fig. 8d and e). In contrast, PVT1 overexpression enhanced the level of PIK3C3 bound to ATG14 and BECN1 both with and without gemcitabine treatment (Fig. 8f). Moreover, the increase in the interaction between BECN1 and PIK3C3 induced by PVT1 overexpression was inhibited by co-transfection with miR-619-5p (Fig. 8g). Furthermore, we also found that gemcitabine treatment significantly increased the interaction between PIK3C3 and ATG14 or BECN1 (Fig. 8f and g). The kinase activity of the PtdIns3K-C1 complex is negatively regulated by Bcl2 family proteins, which bind the BH3 domain of BECN1 [11]. Therefore, the effects of PVT1 on the interaction between BECN1 and Bcl2 were further investigated. The results revealed less Bcl2 bound to BECN1 upon PVT1 overexpression with and without gemcitabine treatment (Fig. 8h). Additionally, we further investigated the effect of miR-619-5p on assembly of the PtdIns3K-C1 complex, and the results revealed that the interaction between BECN1 and PIK3C3 was inhibited by miR-619-5p overexpression (Fig. 8i). PtdIns3P in omegasomes produced by class III PtdIns3K is crucial for autophagy initiation [32]. To determine whether PVT1 directly modulates the activity of the PtdIns3K-C1 complex, a PIK3C3 kinase activity assay was performed. After an immunoprecipitation (IP) assay in PANC-1 and ASPC-1 cells transfected with PVT1 overexpression plasmid with or without gemcitabine treatment with anti-ATG14 antibody was carried out, we incubated the anti-ATG14 immunoprecipitate with phosphatidylinositol as the substrate and conducted quantitative ELISA. The results revealed that ATG14-linked PtdIns3K kinase activity was dramatically increased after PVT1 overexpression and decreased after miR-619-5p transfection both with and without gemcitabine treatment (Fig. 8j-l). ZFYVE1 is widely used as a marker of omegasomes, which are formed by phagophore-associated PtdIns3P-enriched endoplasmic reticulum (ER) membranes,and a downstream effector of PtdIns3K [33]. Immunofluorescence analysis indicated that PVT1 overexpression in PANC-1 cells led to an increase and miR-619-5p mimics led to a decreasing number of GFP-ZFYVE1 puncta with or without gemcitabine treatment (Fig. 8m and n). These data indicate that the PVT1-ATG14 interaction is essential for the activation of ATG14-dependent class III PtdIns3K activity and assembly of the PtdIns3K-C1 complex.

### miR-619-5p reversed the effects of PVT1 on gemcitabine resistance in pancreatic cancer cells

**Fig. 9:**
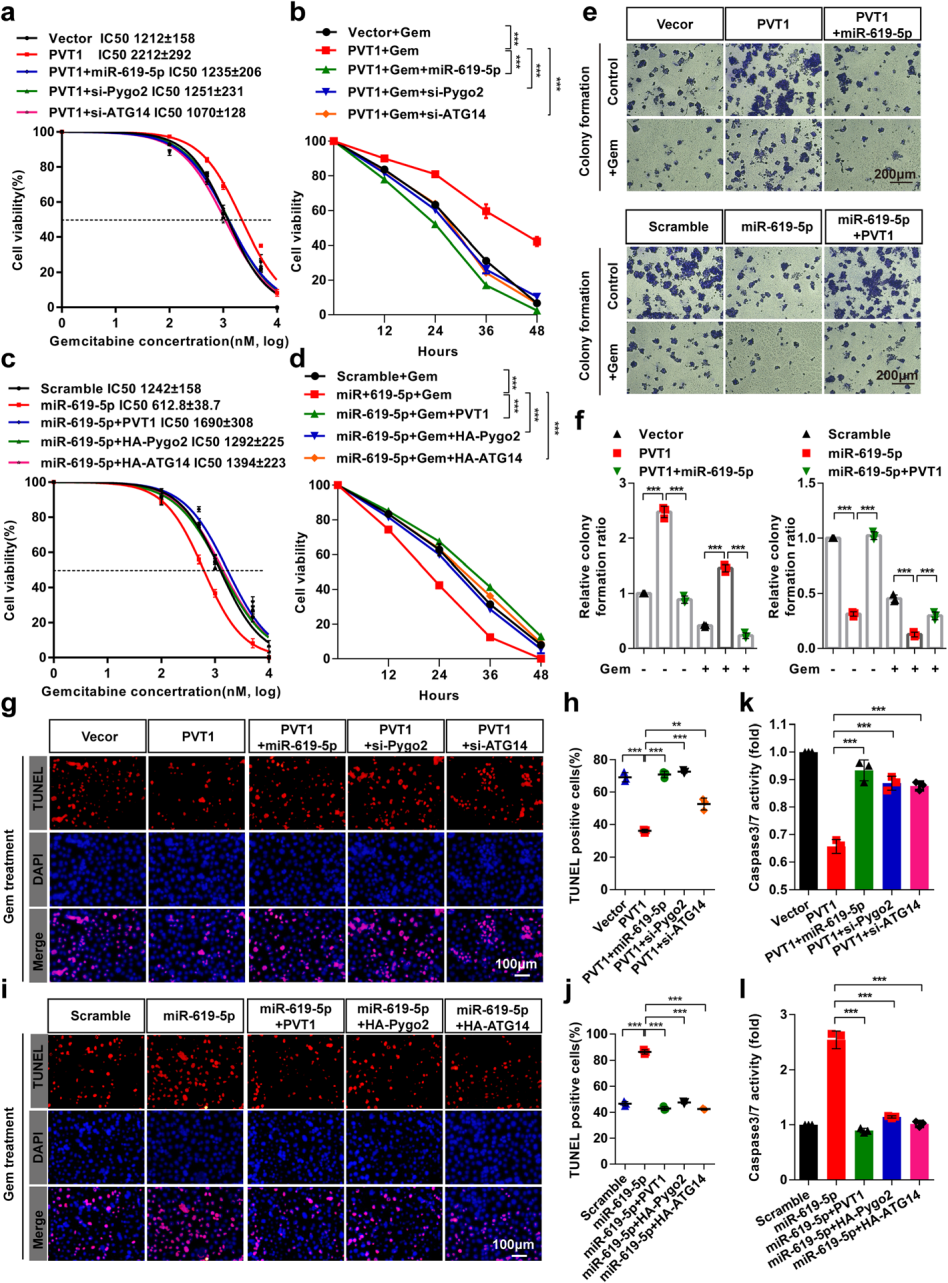
miR-619-5p reverse the effects of PVT1 on gemcitabine resistant in pancreatic cancer cells. **a**-**d** PANC-1 cells were co-transfected with the indicated vectors/siRNAs/miR-619-5p and treated with gemcitabine at different concentrations for 48 h, after which cell viability was measured by MTT assay. **e** and **f** Colony formation assays were performed in PANC-1 cells after co-transfection with PVT1 and/or miR-619-5p mimics. **g-j** PANC-1 cells were co-transfected with the indicated vectors/siRNAs/miR-619-5p and treated with gemcitabine for 48 h, after which the apoptotic cells were analyzed by TUNEL assay, and the number of TUNEL-positive cells was quantified. Scale bars: 100 μm. **k** and **l** The caspase-3/7 activities of PANC-1 cells co-transfected with the indicated vectors/siRNAs/miR-619-5p and treated with gemcitabine were measured with a Caspase-Glo® 3/7 assay kit. Data were represented as mean ± SD, *P < 0.05; **P < 0.01; ***P < 0.001

To investigate the biological role of the PVT1/miR-619-5p axis in human pancreatic cancer gemcitabine resistance, we assessed the viability of PANC-1 cells treated with gemcitabine at different concentrations for different durations. The results showed that co-transfection of PVT1 and miR-619-5p decreased the gemcitabine IC50 value compared with PVT1 overexpression alone (Fig. 9a). Similarly, co-transfection of PVT1 and either Pygo2 siRNA or ATG14 siRNA significantly decreased the gemcitabine IC50 value (Fig. 9a). Moreover, co-transfection of PVT1 with either miR-619-5p, Pygo2 siRNA or ATG14 siRNA significantly decreased cell viability compared with that in cells overexpressing PVT1 in a time-dependent manner (Fig. 9b). We also found that miR-619-5p significantly decreased cell viability and the gemcitabine IC50 value, while co-expression with PVT1, Pygo2 or ATG14 restored these change in cell viability and the gemcitabine IC50 value (Fig. 9c and d). Colony formation activity after PVT1 or miR-619-5p overexpression was assessed, and the results revealed that PVT1 overexpression increased the colony formation activity of PANC-1 cells both with and without gemcitabine treatment; this increase was restored by the co-expression of miR-619-5p mimics. In contrast, the decreased colony formation activity of PANC-1 cells induced by miR-619-5p mimics overexpression was restored by PVT1 overexpressing (Fig. 9e and f). Furthermore, we investigated alteration of the apoptosis rate of pancreatic cancer cells induced by gemcitabine after PVT1 and/or miR-619-5p overexpression. To that end, TUNEL assay was performed and the results showed that co-expression of miR-619-5p, Pygo2 siRNA or ATG14 siRNA significantly restored the number of TUNEL-positive cells compared with that among PVT1 transfected cells (Fig. 9g and h). MiR-619-5p mimics significantly enhanced the number of TUNEL-positive cells compared with that among control cells, and this increase was reversed by co-expression with PVT1, Pygo2 or ATG14 (Fig. 9i and j). Additionally, the caspase 3/7 activity assays showed similar results (Fig. 9k and l). These data indicated that miR-619-5p reversed the effects of PVT1 on gemcitabine resistance in pancreatic cancer cells.

## Discussion

A growing body of research has illuminated that dysregulated lncRNAs may have critical regulatory functions in diverse processes, including cancer initiation and progression [34, 35]. Recent evidence of the roles of lncRNAs in cancer chemoresistance, such as the role of the lncRNA MALAT1 in cisplatin [36] and that of linc00173 in adriamycin and etoposide resistance [37], has also added to our knowledge of cancer biology. Gemcitabine is a first-line chemotherapy drug approved for the treatment of advanced pancreatic cancer, either alone or in combination with other chemotherapeutic agents [2]. Moreover, gemcitabine resistance was also reported to correlate with lncRNAs, such as lncRNA LET [38]. Although dysregulation of certain lncRNAs has been demonstrated in chemoresistant cancer, the functional mechanisms of most lncRNAs in human cancers remain undeterminded. In this study, we discovered that the lncRNA PVT1 is up-regulated in gemcitabine resistant human pancreatic cancer cells, and subsequent gain- and loss-of-function experiments revealed that PVT1 promotes gemcitabine resistance in vitro and in vivo.

Reported data regarding lncRNAs support the ceRNA hypothesis, which proposes that transcripts with shared miRNA binding sites compete for posttranscriptional control [39]. To investigate whether PVT1 can act as a ceRNA in pancreatic cancer gemcitabine chemoresistance, we performed bioinformatic analysis, luciferase assays and RNA immunoprecipitation assays with the Ago2 protein, which revealed PVT1 to engage in complementary binding with miR-619-5p, which is related to cisplatin resistance [40]. Moreover, the miR-619-5p target gene Pygo2 was reported in previous studies to activate MDR1 expression and mediate chemoresistance in human glioma cancer and breast cancer via the Wnt/β-catenin pathway [19, 26]. In addition, ATG14 was reported to activate autophagy and overcome insulin resistance in human hepatoma carcinoma cells [41]. Our data indicated that the ectopic expression of PVT1 was sufficient to increase the expression of Pygo2 and ATG14. Furthermore, we demonstrated that miR-619-5p overexpression, Pygo2 depletion or ATG14 knockdown significantly restored changes in chemoresistance and cell proliferation induced by PVT1 overexpression. Taken together, our data demonstrated that PVT1 functions as a ceRNA to regulate the miR-619-5p/Pygo2 and miR-619-5p/ATG14 axes to improve pancreatic cancer chemoresistance.

Increased Wnt/β-catenin pathway activity plays an important role in chemoresistance. For example, inhibition of MASTL in colorectal cancer cells was reported to induce chemosensitivity to 5FU and down-regulate Survivin and Bcl-xL expression through promoting Wnt/β-catenin signaling [42]. C-Myc is a direct target of Wnt/β-catenin signaling involved in the cell cycle, cell growth, differentiation, apoptosis, transformation, genomic instability and angiogenesis [43]. Notably, C-Myc was also reported to confer resistance against 5FU in colorectal cancer cells [44]. β-catenin, a major transcriptional activator of the canonical Wnt/β-catenin signaling pathway, is important for a series of biological processes, including tissue homeostasis and embryonic development, and is involved in various human diseases. USP20 was reported to positively regulate tumorigenesis and chemoresistance through β-catenin stabilization [45]. Moreover, lncRNAs, such as the lncRNA CRNDE, were also found to regulate cancer chemo-resistance by influencing Wnt/β-catenin signaling [46]. Here, we demonstrated that gemcitabine resistance induced by the lncRNA PVT1 was associated with activated Wnt/β-catenin signaling. Furthermore, among the many factors and causes of acquired drug resistance, Wnt/β-catenin signaling induced chemo-resistance was reported to be related to the up-regulated expression of P-glycoprotein (P-gp/ABCB1), which is encoded by the multidrug resistance 1 (MDR1) gene and plays a significant role in drug resistance due to the increased energy-dependent efflux of cytotoxic drugs from cancer cells [47]. We and other researchers have also previously reported that Pygo2, a newly identified Wnt/β-catenin pathway component that was shown by the present data to be up-regulated by PVT1, activated MDR1 expression via the Wnt/β-catenin pathway, and inhibition of Pygo2 expression restored the chemotherapeutic drug sensitivity of human breast cancer and glioma cells [19, 26]. We also showed that PVT1 could up-regulate the level of P-gp through Pygo2 mediated activation of Wnt/β-catenin signaling.

Notably, we identified three binding elements of the β-catenin/TCF4 complex in the promoter region of PVT1 and further demonstrated that PVT1 was significantly up-regulated by Pygo2 mediated activation of Wnt/β-catenin. We therefore have determined the mechanism of a positive feedback loop between Wnt/β-catenin signaling and PVT1 expression under gemcitabine treatment conditions. PVT1 was also reported to be related to cisplatin sensitivity in colorectal cancer [48] and 5FU resistance in gastric cancer [49]. It would be worth identifying whether the positive feedback loop between PVT1 and Wnt/β-catenin signaling also functions in the chemo-resistance in other cancer types.

Autophagy is a key catabolic process that is essential for maintaining cellular homeostasis and survival through the removal and recycling of unwanted cellular material. ATG14 is a central regulatory protein in PtdIns3K-C1(ATG14/BECN1/PIK3C3 complex) that plays an essential role in the initiation of autophagosomal particles [25]. Moreover, the down-regulation of ATG14 by EGR1-miR152 was shown to sensitize ovarian cancer cells to cisplatin-induced apoptosis by inhibiting cytoprotective autophagy [50]. ADRB2 signaling was also reported to negatively regulate autophagy by disrupting the PtdIns3K-C1 complex in an Akt-dependent manner and promoting sorafenib resistance in hepatocellular carcinoma [51]. We also found that PVT1/miR-619-5p could target ATG14 and promote cellular autophagic activity, and pharmacological or genetic inhibition of autophagy activity restored pancreatic cancer gemcitabine sensitivity. While the vast majority of studies on the regulation of autophagy signaling have focused on protein-protein interactions, post-translational modifications and transcriptional control [52], by using online prediction and RNA pull-down and RNA immunoprecipitation assays, we identified that PVT1 specifically binds ATG14. Furthermore, we also demonstrated that PVT1 significantly enhanced assembly of the ptdIns3K-C1 complex and ATG14-dependent class III PtdIns3K activity. Our findings underscore the importance of identifying new roles for lncRNAs in autophagy under cellular stress.

**Fig. 10:**
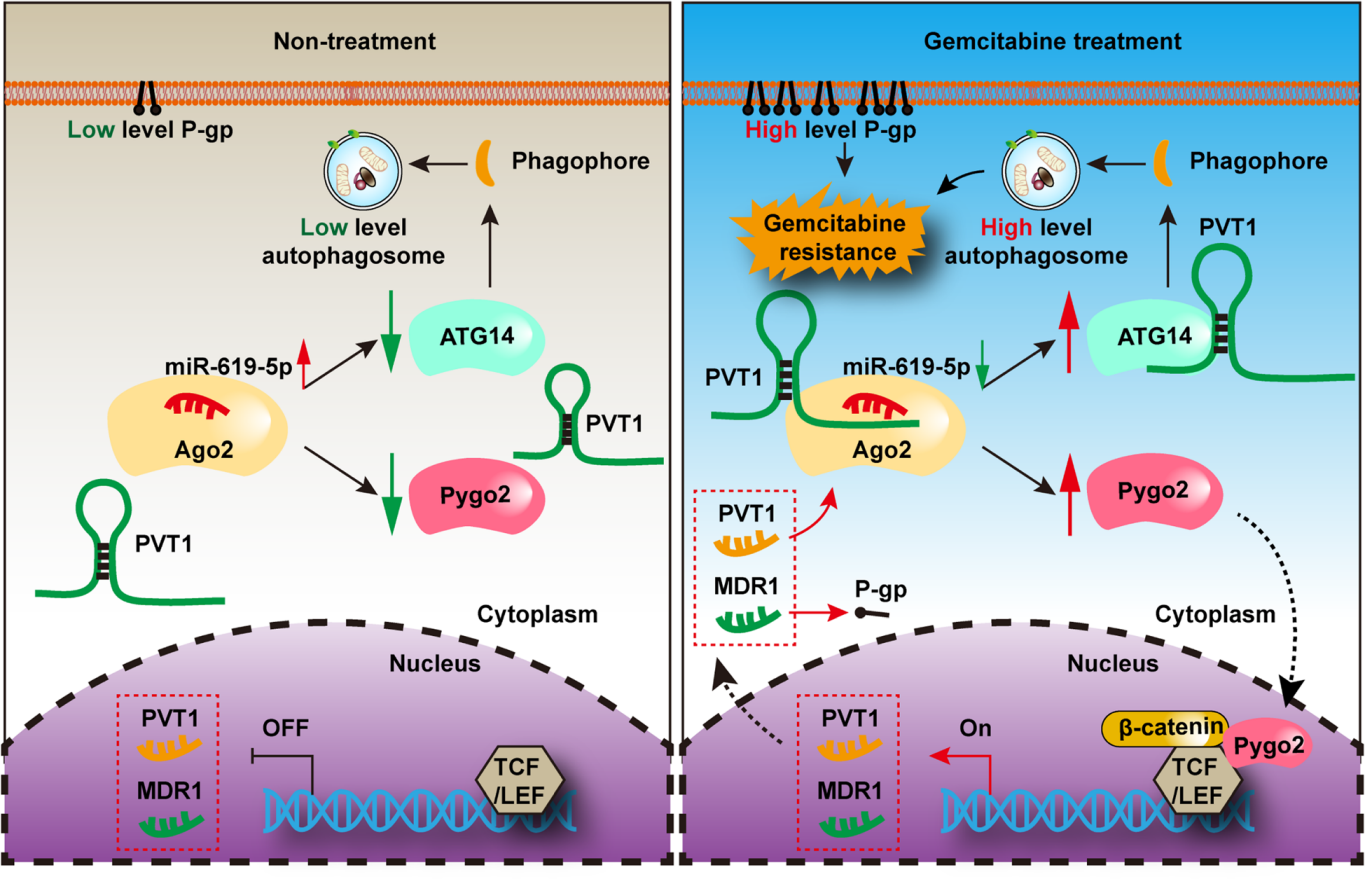
Schematic illustration depicting a proposed model of the molecular mechanism of PVT1 in gemcitabine resistance in human pancreatic cancer

Both Wnt/β-catenin signaling and autophagy were found to benefit from overcoming chemo-resistance in multiple cancers. However, the reported regulatory feedback mechanism between Wnt/β-catenin and autophagy had a suppressive effect [53–55]. The Wnt/β-catenin signaling pathway could suppress autophagosome formation and directly represse the transcription of p62 via TCF4 [53]. In addition, components of Wnt/β-catenin signaling, such as β-catenin [54] and Dishevelled [56] were selectively degraded via the formation of an LC3- or p62-bound complex during nutrient deprivation. The cooperation between Wnt/β-catenin signaling and autophagy has been rarely studied. In the present study, we demonstrated that the novel PVT1/miR-619-5p axis links Wnt/β-catenin signaling and the autophagy pathway through regulating the Pygo2 and ATG14 proteins under gemcitabine treatment conditions, as shown in Fig. 10.

In summary, it is our novel discovery of a positive feedback loop between the lncRNA PVT1 and Wnt/β-catenin signaling involved in human pancreatic cancer gemcitabine resistance. We also demonstrated the mechanism by which lncRNAs regulate autophagy, which occurs through their binding to autophagy-related proteins. We also determined that cooperation between autophagy and Wnt/β-catenin signaling overcomes chemotherapeutic stress.

## Conclusion

Our findings provide insight into PVT1 to be a prognostic marker for pancreatic cancer chemosensitivity, as well as in the development of novel treatment against human pancreatic cancer.

## Supplementary information

**Additional file 1: Figure S1.** PVT1 promotes pancreatic cancer cell viability and inhibit cell apoptosis. **FigureS2.** PVT1 enhances the expression of Wnt/β-catenin target gene. **FigureS3.** PVT1 transcription was enhanced by Wnt/β-catenin signaling pathway. **FigureS4.** The distribution of PVT1 in PANC-1 and ASPC-1 cells

## Data Availability

All data generated or analyzed during this study are included within the article.

## Abbreviations

PVT1: Plasmacytoma variant translocation 1
Pygo2: Pygopus2
ATG14: Autophagy related gene 14; Gemcitabine (2′,2′-difluoro deoxycytidine (dFdC))
LncRNA: Long non-coding RNA
PDAC: Pancreatic ductal epithelial carcinoma
AMPK: Adenosine 5′-monophosphate (AMP)-activated protein kinase
mTOR: Mechanistic target of rapamycin kinase (mTOR)
TUNEL: Terminal dexynucleotidyl transferase (TdT)-mediated dUTP nick end labeling
RIP: RNA-binding protein immunoprecipitation

## References

[1] Siegel RL, Miller KD, Jemal A (2018). Cancer statistics, 2018. CA Cancer J Clin.

[2] Rothenberg ML (1996). New developments in chemotherapy for patients with advanced pancreatic cancer. Oncology (Williston Park).

[3] Conroy T, Hammel P, Hebbar M, Ben Abdelghani M, Wei AC, Raoul JL, Chone L, Francois E, Artru P, Biagi JJ (2018). FOLFIRINOX or gemcitabine as adjuvant therapy for pancreatic Cancer. N Engl J Med.

[4] Ghosal S, Das S, Chakrabarti J (2013). Long noncoding RNAs: new players in the molecular mechanism for maintenance and differentiation of pluripotent stem cells. Stem Cells Dev.

[5] Zhang A, Zhao JC, Kim J, Fong KW, Yang YA, Chakravarti D, Mo YY, Yu J (2015). LncRNA HOTAIR enhances the androgen-receptor-mediated transcriptional program and drives castration-resistant prostate Cancer. Cell Rep.

[6] Wan L, Sun M, Liu GJ, Wei CC, Zhang EB, Kong R, Xu TP, Huang MD, Wang ZX (2016). Long noncoding RNA PVT1 promotes non-small cell lung Cancer cell proliferation through epigenetically regulating LATS2 expression. Mol Cancer Ther.

[7] Zhang XW, Bu P, Liu L, Zhang XZ, Li J (2015). Overexpression of long non-coding RNA PVT1 in gastric cancer cells promotes the development of multidrug resistance. Biochem Biophys Res Commun.

[8] You L, Chang D, Du HZ, Zhao YP (2011). Genome-wide screen identifies PVT1 as a regulator of gemcitabine sensitivity in human pancreatic cancer cells. Biochem Biophys Res Commun.

[9] Kim J, Kim YC, Fang C, Russell RC, Kim JH, Fan W, Liu R, Zhong Q, Guan KL (2013). Differential regulation of distinct Vps34 complexes by AMPK in nutrient stress and autophagy. Cell..

[10] Hosokawa N, Hara T, Kaizuka T, Kishi C, Takamura A, Miura Y, Iemura S, Natsume T, Takehana K, Yamada N (2009). Nutrient-dependent mTORC1 association with the ULK1-Atg13-FIP200 complex required for autophagy. Mol Biol Cell.

[11] Zhou C, Qian X, Hu M, Zhang R, Liu N, Huang Y, Yang J, Zhang J, Bai H, Yang Y, et al. STYK1 promotes autophagy through enhancing the assembly of autophagy-specific class III phosphatidylinositol 3-kinase complex I. Autophagy. 2019;7:1–21. 10.1080/15548627.2019.1687212 PMC8386619 31696776

[12] Levy JMM, Towers CG, Thorburn A (2017). Targeting autophagy in cancer. Nat Rev Cancer.

[13] Yu T, Guo F, Yu Y, Sun T, Ma D, Han J, Qian Y, Kryczek I, Sun D, Nagarsheth N (2017). Fusobacterium nucleatum promotes chemoresistance to colorectal cancer by modulating autophagy. Cell.

[14] Larson-Casey JL, Deshane JS, Ryan AJ, Thannickal VJ, Carter AB (2016). Macrophage Akt1 kinase-mediated Mitophagy modulates apoptosis resistance and pulmonary fibrosis. Immunity..

[15] Karasic TB, O'Hara MH, Loaiza-Bonilla A, Reiss KA, Teitelbaum UR, Borazanci E, De Jesus-Acosta A, Redlinger C, Burrell JA, Laheru DA (2019). Effect of gemcitabine and nab-paclitaxel with or without Hydroxychloroquine on patients with advanced pancreatic Cancer: a phase 2 randomized clinical trial. JAMA Oncol.

[16] Ferrarelli LK (2017). Treating WNT-driven colorectal cancer. Science..

[17] Huang M, Zhang D, Wu JY, Xing K, Yeo E, Li C, Zhang L, Holland E, Yao L, Qin L (2020). Wnt-mediated endothelial transformation into mesenchymal stem cell-like cells induces chemoresistance in glioblastoma. Sci Transl Med.

[18] Wickstrom M, Dyberg C, Milosevic J, Einvik C, Calero R, Sveinbjornsson B, Sanden E, Darabi A, Siesjo P, Kool M (2015). Wnt/beta-catenin pathway regulates MGMT gene expression in cancer and inhibition of Wnt signalling prevents chemoresistance. Nat Commun.

[19] Zhou C, Cheng H, Qin W, Zhang Y, Xiong H, Yang J, Huang H, Wang Y, Chen XZ, Tang J (2017). Pygopus2 inhibits the efficacy of paclitaxel-induced apoptosis and induces multidrug resistance in human glioma cells. Oncotarget..

[20] Livak KJ, Schmittgen TD (2001). Analysis of relative gene expression data using real-time quantitative PCR and the 2(−Delta Delta C(T)) method. Methods..

[21] Zhou C, Yu J, Wang M, Yang J, Xiong H, Huang H, Wu D, Hu S, Wang Y, Chen XZ, Tang J (2017). Identification of glycerol-3-phosphate dehydrogenase 1 as a tumour suppressor in human breast cancer. Oncotarget..

[22] Zhou C, Zhang Y, Dai J, Zhou M, Liu M, Wang Y, Chen XZ, Tang J (2016). Pygo2 functions as a prognostic factor for glioma due to its up-regulation of H3K4me3 and promotion of MLL1/MLL2 complex recruitment. Sci Rep.

[23] Zhou C, Wang M, Zhou L, Zhang Y, Liu W, Qin W, He R, Lu Y, Wang Y, Chen XZ, Tang J (2016). Prognostic significance of PLIN1 expression in human breast cancer. Oncotarget..

[24] Zhou C, Wang M, Yang J, Xiong H, Wang Y, Tang J (2019). Integral membrane protein 2A inhibits cell growth in human breast cancer via enhancing autophagy induction. Cell Commun Signal.

[25] Klionsky DJ, Abdelmohsen K, Abe A, Abedin MJ, Abeliovich H, Acevedo Arozena A, Adachi H, Adams CM, Adams PD, Adeli K (2016). Guidelines for the use and interpretation of assays for monitoring autophagy (3rd edition). Autophagy..

[26] Zhang ZM, Wu JF, Luo QC, Liu QF, Wu QW, Ye GD, She HQ, Li BA (2016). Pygo2 activates MDR1 expression and mediates chemoresistance in breast cancer via the Wnt/beta-catenin pathway. Oncogene..

[27] Yamada T, Takaoka AS, Naishiro Y, Hayashi R, Maruyama K, Maesawa C, Ochiai A, Hirohashi S (2000). Transactivation of the multidrug resistance 1 gene by T-cell factor 4/beta-catenin complex in early colorectal carcinogenesis. Cancer Res.

[28] Fornes O, Castro-Mondragon JA, Khan A, van der Lee R, Zhang X, Richmond PA, Modi BP, Correard S, Gheorghe M, Baranašić D (2020). JASPAR 2020: update of the open-access database of transcription factor binding profiles. Nucleic Acids Res.

[29] Gutschner T, Diederichs S (2012). The hallmarks of cancer: a long non-coding RNA point of view. RNA Biol.

[30] Keihani S, Kluever V, Mandad S, Bansal V, Rahman R, Fritsch E, Gomes LC, Gartner A, Kugler S, Urlaub H (2019). The long noncoding RNA neuroLNC regulates presynaptic activity by interacting with the neurodegeneration-associated protein TDP-43. Sci Adv.

[31] Muppirala UK, Honavar VG, Dobbs D (2011). Predicting RNA-protein interactions using only sequence information. BMC Bioinformatics.

[32] Zhang D, Wang W, Sun X, Xu D, Wang C, Zhang Q, Wang H, Luo W, Chen Y, Chen H, Liu Z (2016). AMPK regulates autophagy by phosphorylating BECN1 at threonine 388. Autophagy..

[33] Klionsky DJ, Emr SD (2000). Autophagy as a regulated pathway of cellular degradation. Science..

[34] Peng WX, Koirala P, Mo YY (2017). LncRNA-mediated regulation of cell signaling in cancer. Oncogene..

[35] Munschauer M, Nguyen CT, Sirokman K, Hartigan CR, Hogstrom L, Engreitz JM, Ulirsch JC, Fulco CP, Subramanian V, Chen J (2018). Publisher correction: the NORAD lncRNA assembles a topoisomerase complex critical for genome stability. Nature..

[36] Zhang YF, Li CS, Zhou Y, Lu XH (2020). Propofol facilitates cisplatin sensitivity via lncRNA MALAT1/miR-30e/ATG5 axis through suppressing autophagy in gastric cancer. Life Sci.

[37] Zeng F, Wang Q, Wang S, Liang S, Huang W, Guo Y, Peng J, Li M, Zhu W, Guo L (2020). Linc00173 promotes chemoresistance and progression of small cell lung cancer by sponging miR-218 to regulate Etk expression. Oncogene..

[38] Zhuang J, Shen L, Yang L, Huang X, Lu Q, Cui Y, Zheng X, Zhao X, Zhang D, Huang R (2017). TGFbeta1 promotes gemcitabine resistance through regulating the LncRNA-LET/NF90/miR-145 signaling Axis in bladder Cancer. Theranostics..

[39] Thomson DW, Dinger ME (2016). Endogenous microRNA sponges: evidence and controversy. Nat Rev Genet.

[40] Zhang J, Sun W, Ren C, Kong X, Yan W, Chen X (2019). A PolH transcript with a short 3'UTR enhances PolH expression and mediates Cisplatin resistance. Cancer Res.

[41] Li B, Wu X, Chen H, Zhuang C, Zhang Z, Yao S, Cai D, Ning G, Su Q (2018). miR199a-5p inhibits hepatic insulin sensitivity via suppression of ATG14-mediated autophagy. Cell Death Dis.

[42] Uppada SB, Gowrikumar S, Ahmad R, Kumar B, Szeglin B, Chen X, Smith JJ, Batra SK, Singh AB, Dhawan P (2018). MASTL induces Colon Cancer progression and Chemoresistance by promoting Wnt/beta-catenin signaling. Mol Cancer.

[43] Oster SK, Ho CS, Soucie EL, Penn LZ (2002). The myc oncogene: MarvelouslY complex. Adv Cancer Res.

[44] Kugimiya N, Nishimoto A, Hosoyama T, Ueno K, Enoki T, Li TS, Hamano K (2015). The c-MYC-ABCB5 axis plays a pivotal role in 5-fluorouracil resistance in human colon cancer cells. J Cell Mol Med.

[45] Wu C, Luo K, Zhao F, Yin P, Song Y, Deng M, Huang J, Chen Y, Li L, Lee S (2018). USP20 positively regulates tumorigenesis and chemoresistance through beta-catenin stabilization. Cell Death Differ.

[46] Han P, Li JW, Zhang BM, Lv JC, Li YM, Gu XY, Yu ZW, Jia YH, Bai XF, Li L (2017). The lncRNA CRNDE promotes colorectal cancer cell proliferation and chemoresistance via miR-181a-5p-mediated regulation of Wnt/beta-catenin signaling. Mol Cancer.

[47] Chen Z, Pan T, Jiang D, Jin L, Geng Y, Feng X, Shen A, Zhang L (2020). The lncRNA-GAS5/miR-221-3p/DKK2 Axis modulates ABCB1-mediated Adriamycin resistance of breast Cancer via the Wnt/beta-catenin signaling pathway. Mol Ther Nucleic Acids.

[48] Ping G, Xiong W, Zhang L, Li Y, Zhang Y, Zhao Y (2018). Silencing long noncoding RNA PVT1 inhibits tumorigenesis and cisplatin resistance of colorectal cancer. Am J Transl Res.

[49] Du P, Hu C, Qin Y, Zhao J, Patel R, Fu Y, Zhu M, Zhang W, Huang G (2019). LncRNA PVT1 mediates Antiapoptosis and 5-fluorouracil resistance via increasing Bcl2 expression in gastric Cancer. J Oncol.

[50] He J, Yu JJ, Xu Q, Wang L, Zheng JZ, Liu LZ, Jiang BH (2015). Downregulation of ATG14 by EGR1-MIR152 sensitizes ovarian cancer cells to cisplatin-induced apoptosis by inhibiting cyto-protective autophagy. Autophagy..

[51] Wu FQ, Fang T, Yu LX, Lv GS, Lv HW, Liang D, Li T, Wang CZ, Tan YX, Ding J (2016). ADRB2 signaling promotes HCC progression and sorafenib resistance by inhibiting autophagic degradation of HIF1alpha. J Hepatol.

[52] Frankel LB, Lubas M, Lund AH (2017). Emerging connections between RNA and autophagy. Autophagy..

[53] Nager M, Sallan MC, Visa A, Pushparaj C, Santacana M, Macia A, Yeramian A, Canti C, Herreros J (2018). Inhibition of WNT-CTNNB1 signaling upregulates SQSTM1 and sensitizes glioblastoma cells to autophagy blockers. Autophagy..

[54] Petherick KJ, Williams AC, Lane JD, Ordonez-Moran P, Huelsken J, Collard TJ, Smartt HJ, Batson J, Malik K, Paraskeva C, Greenhough A (2013). Autolysosomal beta-catenin degradation regulates Wnt-autophagy-p62 crosstalk. EMBO J.

[55] Liu C, Sun L, Yang J, Liu T, Yang Y, Kim SM, Ou X, Wang Y, Sun L, Zaidi M (2018). FSIP1 regulates autophagy in breast cancer. Proc Natl Acad Sci U S A.

[56] Gao C, Cao W, Bao L, Zuo W, Xie G, Cai T, Fu W, Zhang J, Wu W, Zhang X, Chen YG (2010). Autophagy negatively regulates Wnt signalling by promoting Dishevelled degradation. Nat Cell Biol.

