# Supplementary material for: LncRNA PVT1 promotes gemcitabine resistance of pancreatic cancer via activating Wnt/β-catenin and autophagy pathway through modulating the miR-619-5p/Pygo2 and miR-619-5p/ATG14 axes
Source: Mol Cancer. 2020 Jul 29;19:118. doi: 10.1186/s12943-020-01237-y (PMC7389684; doi:10.1186/s12943-020-01237-y)
Supplement: Supplementary file 1 — Additional file 1: Figure S1. PVT1 promotes pancreatic cancer cell viability and inhibit cell apoptosis. FigureS2. PVT1 enhances the expression of Wnt/β-catenin target gene. FigureS3. PVT1 transcription was enhanced by Wnt/β-catenin signaling pathway. FigureS4. The distribution of PVT1 in PANC-1 and ASPC-1 cells [file 12943_2020_1237_MOESM1_ESM.docx]

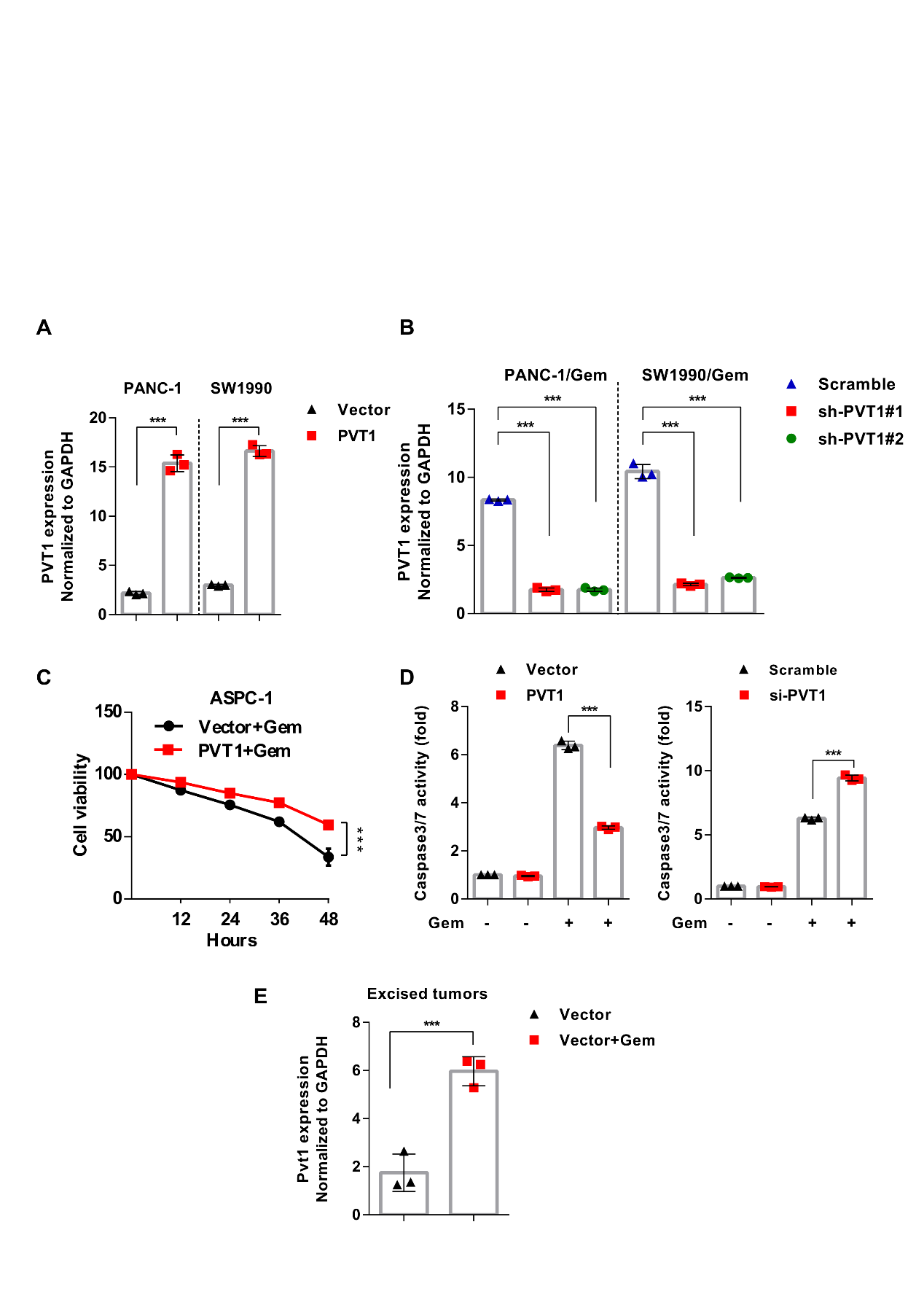


## FigureS1. PVT1 promotes pancreatic cancer cell viability and inhibit cell apoptosis.

(**A and B**) Efficiency of stable overexpression of PVT1 in PANC-1 and SW1990, or knockdown of PVT1 by two shRNAs in PANC-1/Gem and SW1990/Gem cells was verified by real time qPCR. (**C**) ASPC-1 cells were transfected with PVT1 overexpression vector and treated with gemcitabine for different time period as indicated, followed by the measurement of cell viability by MTT assay. (**D**) The caspase-3/7 activities of PANC-1 cells with PVT1 overexpression and knockdown treated with or without gemcitabine were measured with a Caspase-Glo® 3/7 Assay kit. (**E**) Level of PVT1 was assessed by qRT-PCR assay in the excised tumors with or without gemcitabine treatment. Data were represented as mean ± SD, *P < 0.05; **P < 0.01; ***P < 0.001.


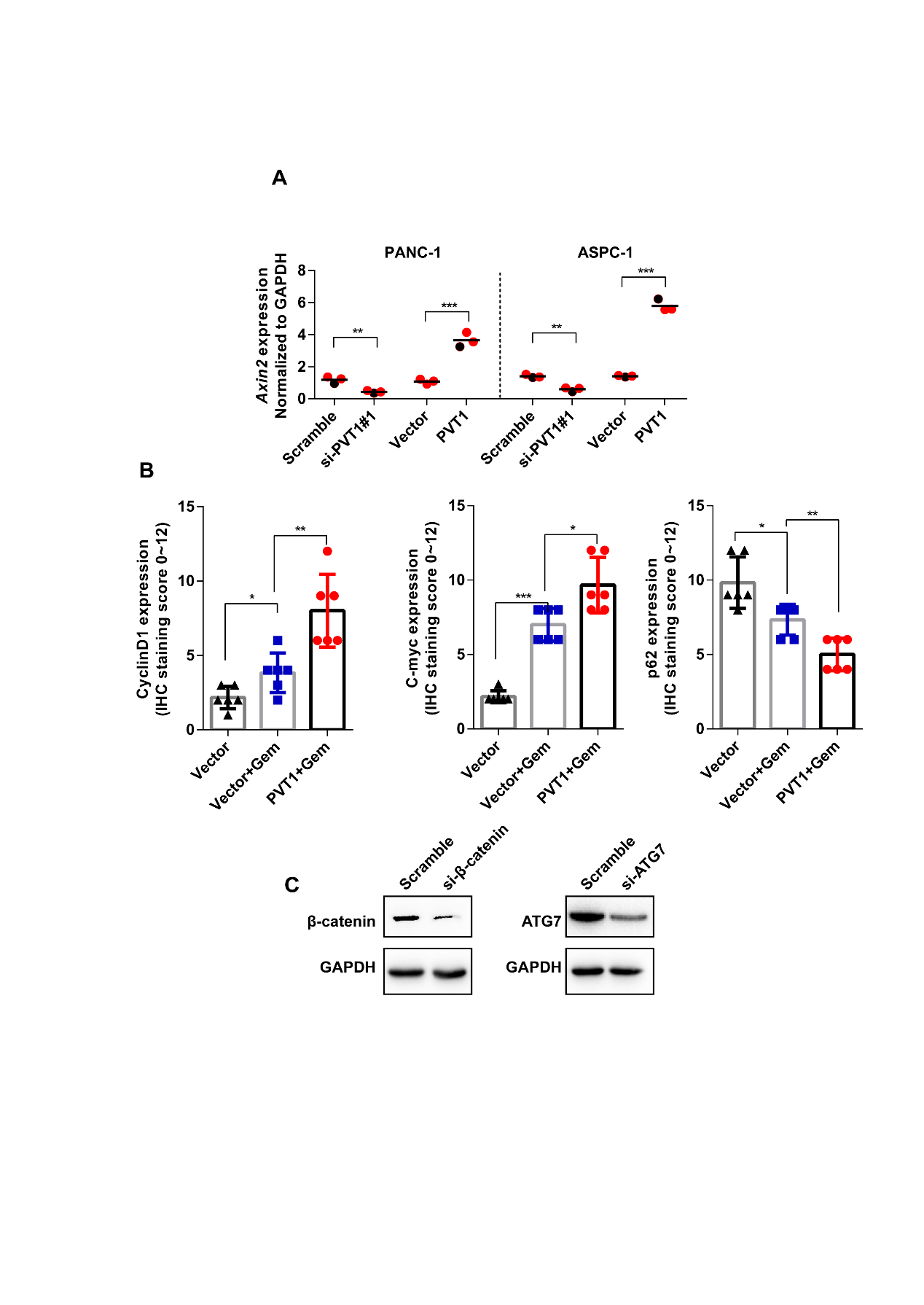


## FigureS2. PVT1 enhances the expression of Wnt/β-catenin target gene.

(**A**) The expression level of *Axin2* in PANC-1 and ASPC-1 transfected with PVT1 overexpression plasmid or PVT1 siRNA were analyzed by real time qRT-PCR. (**B**) The quantification of CyclinD1, C-myc and p62 intensity in excised tumors from nude mice. the intensity of the CyclinD1, C-myc and p62 immunoreaction was divided into five grades depending on the percentage of positive cells (percentage scores): <10% (0), 10–25% (1), 25–50% (2), 50–75 (3), and >75% (4). The intensity of staining was divided into four grades (intensity scores): no staining (0), light brown (1), brown (2), and dark brown (3). The overall staining score was determined by the formula: overall scores = percentage score × intensity score. (**C**) Efficiency of β-catenin/siRNA or ATG7/siRNA in PANC-1 cells was verified by western blotting. Data were represented as mean ± SD, *P < 0.05; **P < 0.01; ***P < 0.001.


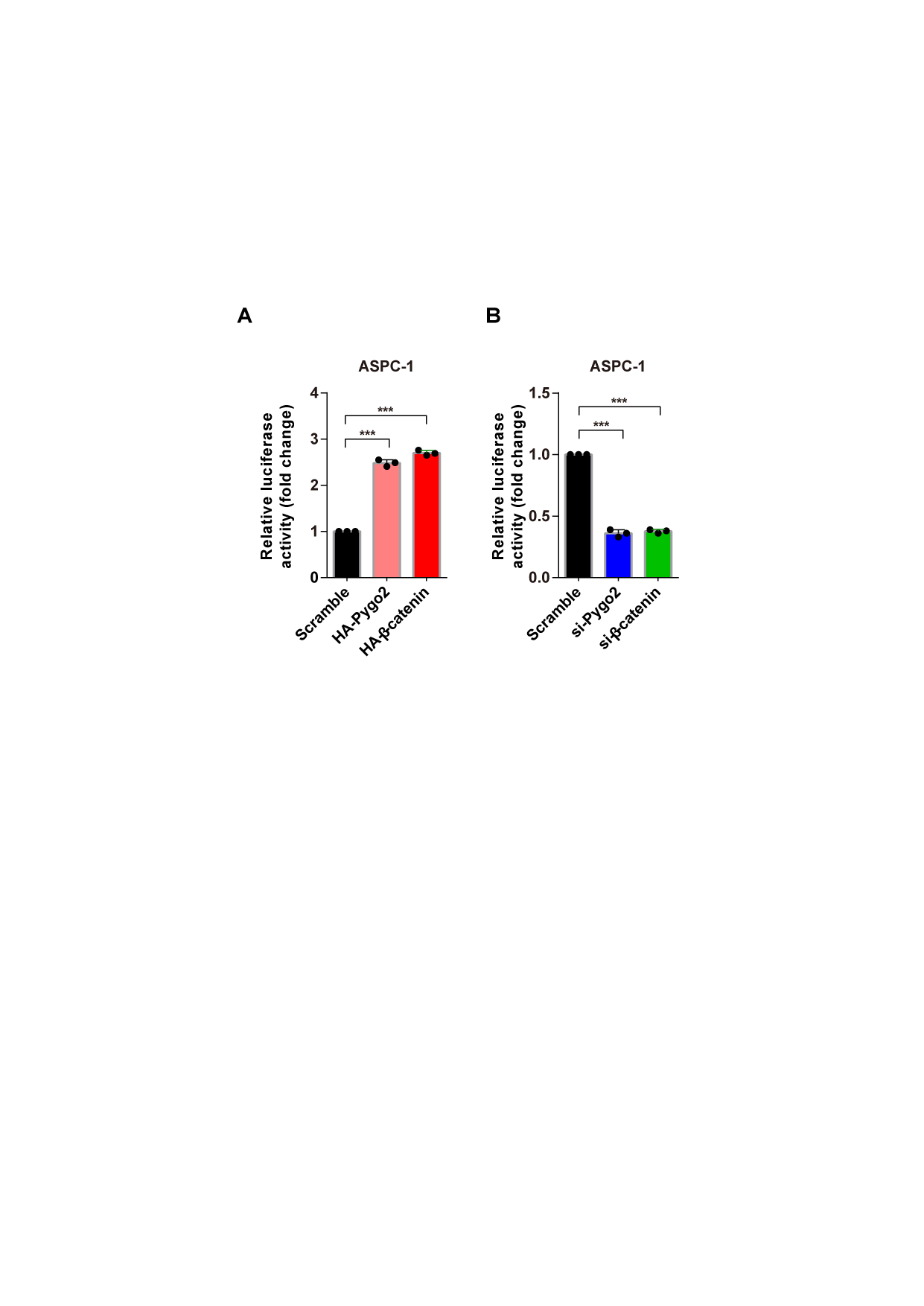


## FigureS3. PVT1 transcription was enhanced by Wnt/β-catenin signaling pathway

(**A and B**) pGL-PVT1 promoter reporter luciferase activity in ASPC-1 cells transfected with β-catenin and Pygo2 overexpression vector or Pygo2/siRNA and β-catenin/siRNA. Data were represented as mean ± SD, *P < 0.05; **P < 0.01; ***P < 0.001.


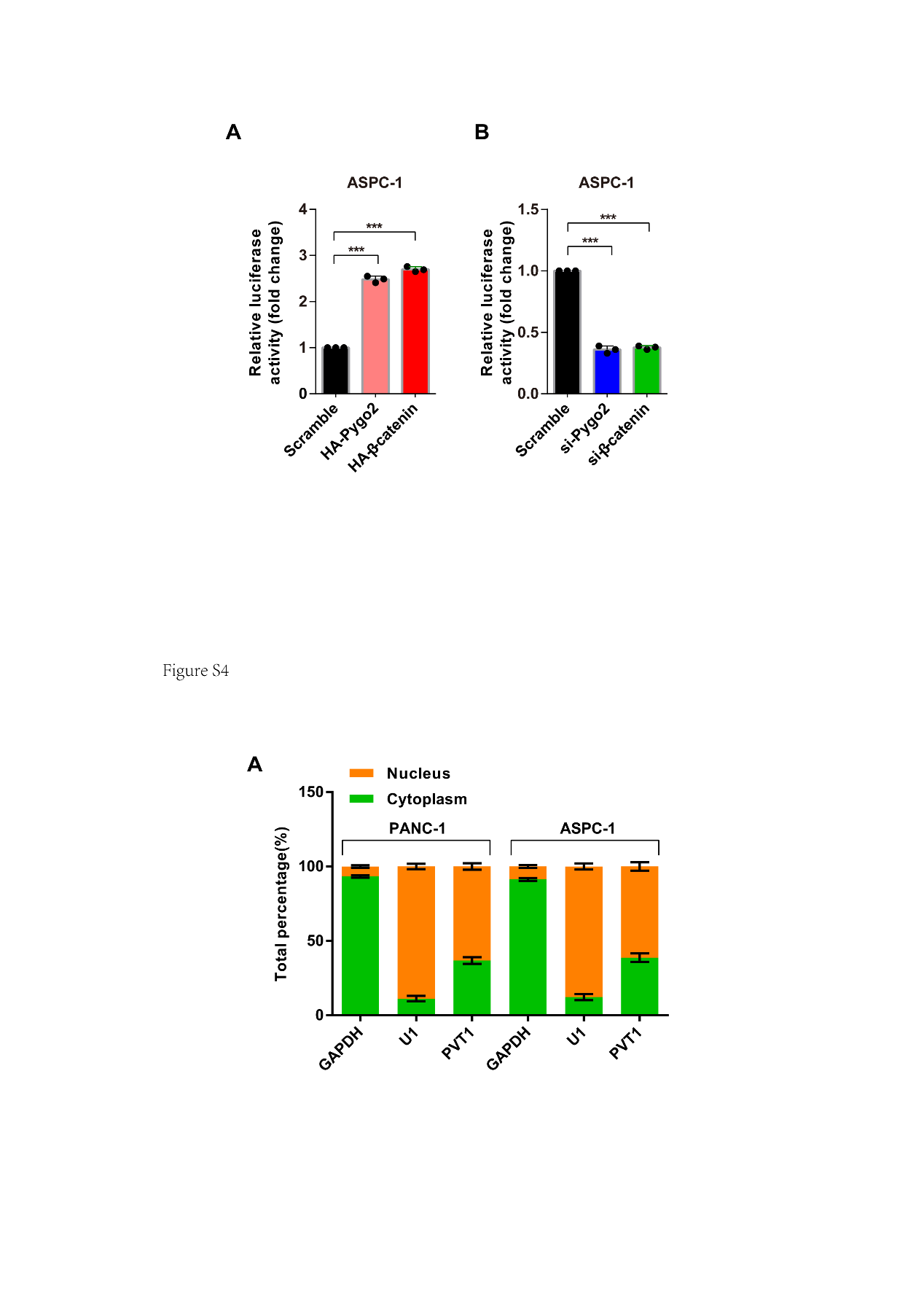


## FigureS4. The distribution of PVT1 in PANC-1 and ASPC-1 cells

(**A**) Nuclear and cytoplasmic fractions of PANC-1 and ASPC-1 cells were subjected to qRT-PCR. U1 and GAPDH were used as nuclear and cytoplasmic positive control, respectively.
